# Current status and future potential of wear-resistant coatings and articulating surfaces for hip and knee implants

**DOI:** 10.1016/j.mtbio.2022.100270

**Published:** 2022-04-30

**Authors:** Charlotte Skjöldebrand, Joanne L. Tipper, Peter Hatto, Michael Bryant, Richard M. Hall, Cecilia Persson

**Affiliations:** a Uppsala University, Department of Materials Science and Engineering, Uppsala, Sweden; b University of Technology Sydney, School of Biomedical Engineering, Sydney, Australia; c Ionbond UK Ltd, Consett, United Kingdom; d University of Leeds, Department of Mechanical Engineering, Leeds, United Kingdom

**Keywords:** Joint implants, Coatings, Surface layers, Ceramics, Biomaterials, Wear resistance

## Abstract

Hip and knee joint replacements are common and largely successful procedures that utilise implants to restore mobility and relieve pain for patients suffering from e.g. osteoarthritis. However, metallic ions and particles released from both the bearing surfaces and non-articulating interfaces, as in modular components, can cause hypersensitivity and local tissue necrosis, while particles originating from a polymer component have been associated with aseptic loosening and osteolysis. Implant coatings have the potential to improve properties compared to both bulk metal and ceramic alternatives. Ceramic coatings have the potential to increase scratch resistance, enhance wettability and reduce wear of the articulating surfaces compared to the metallic substrate, whilst maintaining overall toughness of the implant ensuring a lower risk of catastrophic failure of the device compared to use of a bulk ceramic. Coatings can also act as barriers to inhibit ion release from the underlying material caused by corrosion. This review aims to provide a comprehensive overview of wear-resistant coatings for joint replacements – both those that are in current clinical use as well as those under investigation for future use. While the majority of coatings belong predominantly in the latter group, a few coated implants have been successfully marketed and are available for clinical use in specific applications. Commercially available coatings for implants include titanium nitride (TiN), titanium niobium nitride (TiNbN), oxidized zirconium (OxZr) and zirconium nitride (ZrN) based coatings, whereas current research is focused not only on these, but also on diamond-like-carbon (DLC), silicon nitride (SiN), chromium nitride (CrN) and tantalum-based coatings (TaN and TaO). The coating materials referred to above that are still at the research stage have been shown to be non-cytotoxic and to reduce wear in a laboratory setting. However, the adhesion of implant coatings remains a main area of concern, as poor adhesion can cause delamination and excessive wear. In clinical applications zirconium implant surfaces treated to achieve a zirconium oxide film and TiNbN coated implants have however been proven comparable to traditional cobalt chromium implants with regards to revision numbers. In addition, the chromium ion levels measured in the plasma of patients were lower and allergy symptoms were relieved. Therefore, coated implants could be considered an alternative to uncoated metal implants, in particular for patients with metal hypersensitivity. There have also been unsuccessful introductions to the market, such as DLC coated implants, and therefore this review also attempts to summarize the lessons learnt.

## Graphical abstract

**Figure:**
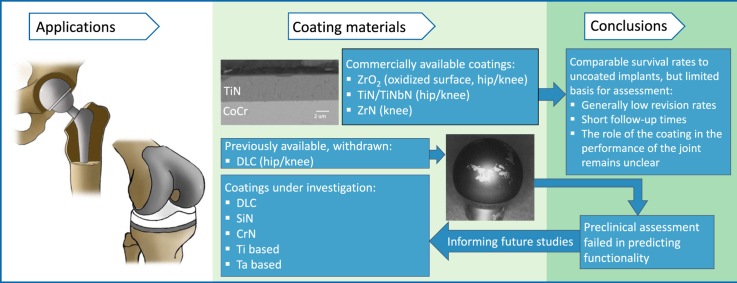

## 1 Introduction

A damaged or diseased joint can cause severe pain and limited mobility, which impairs the quality of life of the afflicted individual. To treat this condition, it might be necessary to replace the joint with an implant in a surgical procedure. Two of these types of implants, namely hip and knee implants, will be the focus of this review. The number of primary, or first time, total hip surgeries performed every year has steadily increased and is predicted to continue to rise [1] as the ageing population increases, and indications are that younger patients can also benefit from these procedures. While hip joint implants have a generally high survival rate of approximately 95% or more at 10 years for all currently used material combinations, metal-on-polyethylene (MoP) being the most common, [[2], [3], [4], [5]]. However, the increasingly active, ageing and younger populations require longer lasting implants and a focus of current research is on prolonging the lifespan of implants to spare patients from further pain and revision surgeries. Similarly, the revision rates for knee implants are reported to range from 4.3% [5] to 5.3% [4] at 10 years.

The primary cause of revision surgery for metal or ceramic hip replacement components paired with polyethylene (PE) is aseptic loosening of the implant (28–51% [[2], [3], [4], [5], [6], [7], [8], [9], [10]]). In these, one of the main causes for this is believed to be the presence of wear debris from the articulating surfaces, mainly polymeric particles. However, it should be noted that PE wear has decreased dramatically since the introduction of highly crosslinked polyethylene (XLPE) 20 years ago [11]. Necrosis, pseudo tumours and pain have also been found to be the cause of revision surgeries particularly in alternative metal on metal bearing systems. These complications are believed to be caused by metallic particulate and soluble (ionic) debris originating from the articulating and non-articulating surfaces of the implant [[12], [13], [14]]. While the particulate debris may originate from any surface of the implant, it is primarily formed at the articulating interface.

The most common materials currently implanted in this context are cobalt chromium alloy (CoCr) and highly crosslinked polyethylene (XLPE) in MoP implants [3,4,9,10], together with a zirconia/alumina ceramic combined with PE (CoP) [4,10]. In these cases, the dominant particulate debris is that of the polymer, which can range between 10 ​nm and 1 ​mm in size. Particles in the size range 0.1–1.0 ​μm are believed to play an important role in the activation of macrophages [15], which might initiate a cascade of reactions that eventually cause wear-induced osteolysis (loss of bone), possibly resulting in implant loosening [[15], [16], [17], [18], [19]]. Metal-on-metal (MoM) hip re-surfacing implants were considered an alternative for young patients, however, the implants experienced high short-term failure rates and an increased rate of revision surgeries [20]. Due to these catastrophic outcomes MoM hip replacements are now rarely used [2,4,5,8]. Debris from MoM implants is generally in the nanoscale range (<100 ​nm) and there is evidence to suggest that metal particles in this size range and ions can cause a variety of biological effects such as hypersensitivity, pseudo-tumours [12,14,[21], [22], [23], [24], [25]] and aseptic lymphocytic vasculitis-associated lesions [13]. In addition high concentrations of cobalt and chromium ions are believed to cause e.g. acute visual and auditory impairment, peripheral neuropathy and cardiomyopathy [26]. It should be noted that the latter are extreme cases, and the incidence of adverse reactions to metal debris has been found to be less than 1.2% for MoM hip implants [27]. The revision per 1000 prosthesis years caused by adverse reactions to particulate debris for MoM was found to be 9.90, which can be compared to 0.19 for MoP [5].

Several different strategies have been explored for reducing wear and the negative biological effects of metal ions and PE wear debris. Among the most noteworthy of these are the improvement of polymer wear properties [11,28] and the development of e.g. textured surfaces [29]. However, this review will focus on the use of ceramic coatings or surface modifications, such as nitriding, to reduce wear and ion release. These aim to combine the advantages of a wear resistant ceramic with the ductility and toughness of a metal. The ceramic coating can also act as a barrier to, and decrease, ion release from the underlying metal [30].

Previous reviews [31,32] have focused on tantalum, graphite-like carbon (GLC) [33,34], diamond-like carbon (DLC) [[35], [36], [37], [38]], titanium nitride based coatings [[39], [40], [41], [42], [43]] and chromium nitride (CrN) [44,45]. However, a number of novel experimental coatings have been reported (e.g. silicon nitride (SiN) [[46], [47], [48], [49], [50], [51], [52], [53]], multilayer structured coatings [42,44,54]) as well as commercially available coatings (oxidized zirconium (OxZr) [55] and titanium nitride (TiN) [56,57]), which are utilised in interfaces of joint prostheses, and currently, there is no comprehensive review available. This review aims to provide readers with a comprehensive overview of coatings that are (i) commercially available and (ii) currently being researched, and to summarize the current status and future potential of such coatings for hip and knee joint implants. We focus on coatings and treatments for articulating surfaces, and as such, other types of coatings, e.g. those for improved bone ingrowth and fixation lie beyond the scope of this review.

## 2 Methodology

**Fig. 1:**
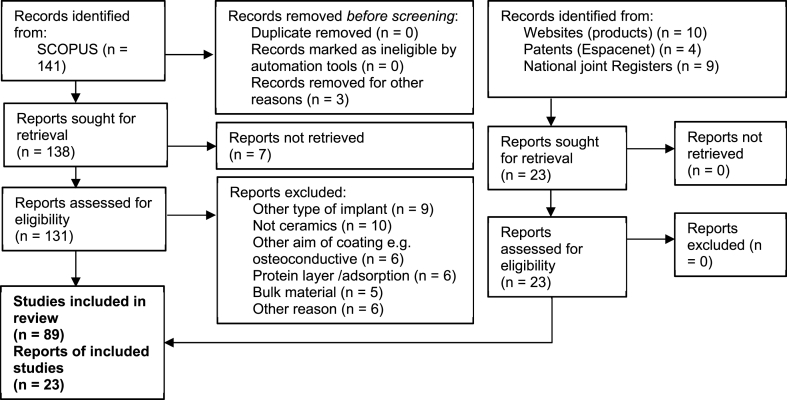
A flow chart showing how the data was collected and treated. The supplementary information contains additional references such as ISO and ASTM standards, studies on background such as biological reactions to debris, and information regarding deposition techniques.

The material in this review is based on published research articles, patents and product information in order to cover all phases from early stage research to products in clinical use. The research articles were found using the database SCOPUS with the primary search terms “wear” AND “coatings OR film” AND “joint implant” for coatings and the search terms “wear” AND “surface modification” AND “joint implant” for surface modifications. An additional search with the terms “biocompatibility” AND “coatings OR film” AND “joint implant” was conducted to include studies regarding the biocompatibility and finally, through references, an additional 6 papers were identified. The patents were found by using the same search terms on Espacenet, and the products were found using Google (with search terms “joint replacement” “products” “coating”). Research papers, patents and products concerning dental implants, osteoinductive/conductive coatings, protein and other biological coatings as well as bulk materials were excluded and the joint types were limited to hip and knee. This left a total number of 89 research papers (27 of them being case reports or revision studies) and 4 patents. Information such as wear rates and surface roughness was compiled in a table that may be found in the supplementary information. Therefore, some of the references are not included in the main paper but can be found in the supplementary information. Fig. 1 shows a flow diagram of the procedures followed in selecting the reports and associated data for inclusion in this review.

## 3 Substrate material and deposition methods

A coating used for joint implants should be hard, wear resistant, corrosion resistant, biocompatible, not release particulates and any particulates that are generated should be biocompatible. In addition, the coating should have a low surface roughness and a good adhesion to the substrate material. Some of these properties are specified in standards such as ISO 7206-2 while others, such as wear resistance, have no specified target value. The important properties and what to consider are specified in Table 1. It should be noted that the table distinguishes between wear resistance and corrosion resistance, however theses are processes that occur synergistically. Typically, these properties are however reported separately.

**Table 1:** The important properties of coatings for joint implants, their evaluation methods and their target profile.

Property	Target profile	Typical method for evaluation and standards
Hardness	A high hardness will help mitigate wear. However, the stiffness should also be considered as the ratio of hardness to Young's modulus gives a measure of the elastic limit in the contact and hence provides an indicator of wear performance.	Nano- or microindentation. Values are given in Pa or Vickers hardness number (HV).
		• *ASTM* E2546-07 [58]
		• *ISO 14577-4* [59]
Wear resistance	A low wear rate is desirable, but actual values will depend on the specific tribological situation and are therefore not specified here. Attention should be paid to the generated wear debris, the size, shape and volume will likely influence the immune response in the final application.	Tribological set-up ranging for pin-on-disc to joint simulators. The resulting wear is measured as specific wear rate (mm^3^/Nm) or mass loss per million cycles (mg/Mc).
		• *EN 1071-12* [60]
		• *EN 1071-13* [61]
		• *ASTM* F732 [62]
		• *ISO 20808* [63]
Corrosion resistance	A coating should protect the underlying metal from corrosion as well as have a low rate of degradation. However, it is also important to consider the character of the particles and ions that inevitably are released.	Measuring open circuit potential (V) and corrosion current (μA).
		• *ASTM* G5 [64]
		• *ISO 16429* [65]
		• *ISO 16773* [66]
Toxicity	Ultimately the coating, and more importantly the ions and wear debris, should not elicit an adverse immune response. The toxicity will depend on the volume of debris or ions, i.e. a dose dependency, and the volume will depend on the wear properties of the coating.	*In vitro* studies using cell lines. Results are given as cell viability.
		• *ISO 10993* [67]
Surface roughness	A smooth surface is necessary to reduce PE wear. An R_a_ value of ≤20 ​nm has been specified for ceramics in ISO 7206-2.	Optical or stylus methods for surface characterization. The most common parameter to report is the average surface roughness, Ra, (nm).
		• *ISO 4287* [68,69]
		• *ISO 4288* [70]
		• *ISO 25178-604* [71]
Adhesion	A coating that adheres well to the substrate is of utmost importance as delamination of the coating could cause excessive wear through the release of abrasive debris. It is important to consider factors such as time and corrosive environments when evaluating the adhesion.	Most common methods are scratch tests, from which critical loads are obtained (N), or Rockwell indentations that are categorized according to a standard.
		• *ISO 26443* [72]
		• *ISO 20502* [73]

These properties are covered in more detail in the supplementary information section.

Ceramic coatings are typically deposited onto a metal implant using a wide range of techniques [[74], [75], [76]]. Possible substrate materials include the metals commonly used as bulk materials for joint implants i.e. CoCr and stainless steel but also titanium. Titanium is not used in articulating surfaces due to its poor wear properties [[77], [78], [79]], but this becomes possible with a coating or surface treatment that improves such properties [75]. The use of titanium as a substrate material could even be advantageous in terms of coating adhesion [80]. It is however important to keep in mind that the coating should be compared with the material it aims to substitute, e.g. in the case of articulating surfaces typically CoCr.

The choice of deposition method or surface treatment will have a strong influence on the above-mentioned parameters. The most common deposition techniques used for coatings for articulating surfaces can be divided into either physical vapor deposition (PVD) methods [81] or chemical vapor deposition (CVD) methods [82], the former group is more commonly used than the latter in the studies found in this review. These deposition techniques, as well as their advantages and challenges, have been covered in detail in previously published articles [[82], [83], [84], [85], [86]]. PVD techniques include, amongst others, vacuum evaporation, magnetron sputtering (MS), reactive magnetron sputtering (rMS), pulsed laser deposition (PLD) and high-power impulse magnetron sputtering (HiPIMS). They utilise metallic sources that are either evaporated, typically using an electron beam, or sputtered with the help of a plasma to produce a flux of metal atoms/ions which is deposited onto the surface to be coated in the presence of one or more reactive gases to form a layer of ceramic material. Coatings deposited using PVD techniques typically have a low surface roughness, are hard and wear resistant. There are however limitations due to the technique being line-of-sight but these are usually mitigated by manipulation of the substrate during deposition. The second group of methods, CVD, entail exposing the substrate to a mixture of gases that react at high temperature (typically >600 ​°C) to form a ceramic compound, though it is possible to use a plasma to enhance the reactivity of the gas precursors (plasma-enhanced CVD (PECVD)) to reduce the deposition temperature. Using CVD techniques it is possible to grow uniform, well adhering coatings on complicated substrates. However, there are limitations related to e.g. heat resistance of the substrate. Another approach is to treat the surface by e.g. heat or laser in the presence of gases such as nitrogen [87]. This often yields an increased hardness and wear resistance, however the surface roughness is often increased and the techniques are limited to materials that can form suitable ceramic surface layers such as zirconium oxide and titanium nitride.

Another family of surface deposition techniques is thermal spraying in which heated or melted material is sprayed onto a surface [88]. The coating material is either in the form of powder or a filament that is typically melted by flame or plasma [75].

A harder surface can also be achieved by surface treatment processes such as plasma electrolytic oxidation where metals are oxidized through a process similar to anodization but with higher potentials [89].

A technique with future potential is additive manufacturing where it is theoretically possible to manufacture implants with a surface layer with different properties compared to the underlying material. However, the manufactured materials currently require extensive post processing to achieve smooth surfaces suitable for wear-resistant applications [90].

The most important properties relevant to coatings for joint implants, as well as the methods used in their evaluation, are discussed in the supplementary information section. It should be noted however that somewhat different requirements exist depending on joint application, e.g. hip joints may experience more complex movement patterns, as well as more severe edge loading [91] than knee joints [92], and different standards are available to evaluate their performance.

## 4 Commercial coatings

There are currently three types of commercially available coated or surface treated hip and knee joint implants for clinical applications, namely:

• Titanium nitride and titanium niobium nitride (TiN and TiNbN) by various companies including:
  Implantcast [56].
  Cellumed [93].
  OHST medical technology [94].
  Link orthopaedics [95].
  Corin [96,97].
• Zirconium nitride (ZrN):
  Aesculap (B Braun) [98].
• Oxidized zirconium (OxZr)
  Smith and Nephew [99].

TiN coated implants are available with either a Ti6Al4V [100] or a CoCr [101] substrate and the coated surface is usually paired with PE in the articulating surfaces. These implants are aimed for young, active patients, in particular patients with metal sensitivity [102] and studies have shown them to be stable over time [103].

As TiN, ZrN and OxZr are commercially available it is possible to study patient outcomes (Table 2). Since their introduction, long-term follow up studies have found that OxZr implants perform well, with low revision rates [55], comparable to uncoated CoCr implants [104,105] (Table 2). The implant has performed better, with lower rates of corrosion and fretting wear, in comparison to CoCr, as seen in retrieved implants [106]. Taking all these results together Oxinium implants show great promise as a viable option for joint implants, not just for patients suffering from or at risk of developing metal hypersensitivity but also for otherwise healthy patients.

**Table 2:** Revised implants and follow-up studies of coated implants. In the case of revision-retrieved samples one must be aware of the fact that they are failures and may depart from the general performance of the cohort.

Coating	Implant	Product	Number of coated implants	Average time of implantation/follow-up [months]	Revison rate	Reason for revision/retrieval	Key findings	Reference
TiNbN	Hip	–	1	53	n.a.	Aseptic loosening	No signs of metallosis. Coating failure due to insufficient adhesion, corrosion, and involvement of third bodies.	Łapaj et al., 2016 [112]
TiN	Knee	Implantcast ACS and Corin Uniglide	5	16	Retrieval study (100%)	Aseptic loosening: 4	TiN coatings of knee replacements undergo wear and degradation related to presence of third bodies and microscopic defects on their surface.	Łapaj et al., 2020 [113]
						Periprosthetic inflammation: 1		
TiNbN	Knee	–	59	36	0	n.a.	Chromium concentrations in patient plasma increased from 0.25 to 0.75 ​μg/l in the coated TKA group compared with of 0.25–1.30 ​μg/l in the standard TKA group.	Postler et al., 2018 [114]
TiN	Knee	ACS® MB system, Implantcast	25	30.7		Infection: 11 Septic loosening: 4 Ligament instability: 2 Pain: 2 Aseptic loosening: 1 Dislocation: 3 Fracture: 1 Recurrent effusion: 1	TiN provides low wear rates and little surface damage	Fabry et al., 2017 [115]
					Retrieval study (100%)			
TiN	Knee	B-P™ knee system	1031	46	2.2%	Malpositioning of tibial component: 6 Traumatic event: 5 Pain (Isolated patellofemoral, instability and arthrofibrosis): 11 Infection: 2	TiN coated total knee replacements perform up to par with conventional implants, but does not solve the problem with residual pain.	Breugem et al., 2017 [116]
TiN	Knee	ACS® Basic, Implantcast	51	62	5.8%	Aseptic loosening: 2 Layer spinout: 1	No difference between coated and conventional implants.	van Hove et al., 2015 [117]
TiN	Knee	B-P™ knee system	61	33	n.a.	n.a.	TiN coated implants showed a high degree of satisfaction and less intraoperative bone mass removal compared to NexGen-LPS implants.	Moon et al., 2012 [118]
TiN	Hip	B-P™ Integrated Hip system, Endotec	1	12	n.a.	Unrelated causes	Well-functioning implant, close future monitoring needed	Harman et al., 1997 [111]
								
OxZr	Hip	Oxinium, Smith & Nephew	3	7	Retrieval study (100%)	Dislocation	The Zr substrate may deform in the case of dislocation because of its low hardness	Kop et al., 2007 [110]
OxZr	Hip	Oxinium, Smith & Nephew	1	0.5	Retrieval study (100%)	Dislocation	Damage to the ZrO_2_ coating and exposed Zr substrate after dislocation	Evangelista et al., 2007 [107]
OxZr	Hip	Oxinium, Smith & Nephew	56	30	n.a.	Not revised	2D wear analysis of radiographs show reduced wear of oxinium femoral heads compared to CoCr	Garvin et al., 2009 [55]
OxZr	Knee	Genesis II, Smith & Nephew	98	74.4	0%	Not revised	Survivorship of 98.7% at 7 years	Innocenti et al., 2010 [119]
OxZr	Knee	Oxinium, Smith & Nephew	11	18.5	Retrieval study (100%)	Stiffness: 7 Infection: 1 Instability: 1 Mal-positioning: 2 Loosening: 1	Lower damage of both the OxZr femoral component and PE tibial component for Oxinium compared to CoCr.	Heyse et al., 2011 [120]
OxZr	Hip	Oxinium, Smith & Nephew	1	48 ​h	Retrieval study (100%)	Correction of leg length discrepancy	Extensive PE wear, loss of the ZrOx layer and Ti transfer from the acetabular shell.	McCalden et al., 2011 [108]
OxZr	Hip	Oxinium, Smith & Nephew	60	24	n.a.	Not revised	Further follow-up needed to be able to discern differences between CoCr and Oxinium	Kadar et al., 2011 [105]
OxZr	Knee	Oxinium, Smith & Nephew	16	16.4	Retrieval study (100%)	Stiffness, infection, instability and dislocation	Wear comparable to conventional MoP implants	Heyse et al., 2011 [121]
OxZr	Knee	Oxinium, Smith & Nephew	109	70.8	n.a.	Not revised	OxZr is an attractive option for patients with metal sensitivity and patients in risk of high rates of wear (due to young age or high activity levels).	Hofer et al., 2014 [122]
OxZr	Knee	Oxinium, Smith & Nephew	98	135.6	2.3%	Loosening.	Survival rate of OxZr of 97.8% at 10 years.	Innocenti et al., 2014 [123]
OxZr	Knee	Oxinium, Smith & Nephew	71	62	n.a.	No revision for loosening.	OxZr comparable to the standard knee prosthesis but further follow up needed.	Park et al., 2014 [124]
OxZr	Hip	Oxinium, Smith & Nephew	60	60	n.a.	Not revised.	Radiostereometric analysis was used to determine OxZr was comparable to, but not better than, CoCr.	Jonsson et al., 2015 [104]
OxZr	Hip	Oxinium, Smith & Nephew	11	8.64∗	Retrieval study (100%)	Aseptic loosening: 14∗ PE wear/osteolysis: 17∗ Fracture: 1∗ Instability: 5∗ Infection: 10∗ Malposition: 4∗ Multiple reasons: 1∗	No difference between OxZr and CoCr femoral heads with regards to fretting and corrosion, however bulk ceramic performed better than both OxZr and CoCr.	Tan et al., 2016 [106]
				∗ *Both bulk ceramic and OxZr*		∗ *Both bulk ceramic and OxZr*		
OxZr	Hip	Oxinium, Smith & Nephew	3	57.3	Retrieval study (100%)	Pain, hip squeak and limited movement.	Misuse of Oxinium heads (pairing Oxinium femoral heads with alumina liners) caused damage to the coated surface and high wear rates.	Ozden et el. 2017 [109]
OxZr	Knee	Oxinium, Smith & Nephew	5969	144	7.7%	Infection, loosening or lysis, patellofemoral pain, pain and instability the most common reasons for revision.	The cumulative revision risk was higher for Oxinium than CoCr (7.7% and 4.8% respectively). Loosening/lysis was the reason for revision in 1.1% of cases.	Vertullo et al., 2017 [125]
OxZr	Knee	Oxinium, Smith & Nephew	10,477	156	0.46%	Infection (the only reason investigated)	Overall same risk of infection for OxZr as CoCr.	Vertullo et al., 2018 [126]
ZrN	Knee	Aesculap	1	18	Not revised	n.a.	The wound healed without complications and the patients eczema as well as the knee pain had disappeared at the last follow up of 18 months.	Thomsen et al., 2011 [127]
ZrN, TiN and TiNbN	Knee	Implantcast, AlphaNorm (now aquired by Corin), Mathys, Link and Aesculap	28	TiN(CoCrMo): 42 TiNbN(CoCrMo): 40.8 ZrN(CoCrMo): 7.8 TiN(Ti6Al4V): 114 TiNbN(Ti6Al4V): 12		Infection: 12 Aseptic loosening: 10 Instability/Luxation: 3 Arthrofibrosis: 1 Periprosthetic fracture: 1 Movement deficit: 1		Herbster et al., 2020 [80]
DLC	Hip	Adamante®, Biomecanique	101	110.4	25.8%	Aseptic loosening: 41 Ossification: 1 Pain: 2 Infection: 1 Implant failure: 1	54% survival for DLC/PE implants at 8.5 years compared to 88.2% for Al_2_O_3_/PE implants. Delamination of the coating caused aggravated wear of the PE liner.	Taeger et al., 2003 [128]

However, there have been cases with both TiN and OxZr in which retrieved implants were reported to exhibit surface damage, including an exposed substrate [[100], [101], [102], [103],^108^]. While the femoral heads in these studies have been paired with a PE liner the oxidized surface has shown damage and metallic transfer likely caused by contact with the metallic shell following dislocations. The authors concluded that Oxinium femoral heads should not be used in patients at risk of joint instability.

## 5 Potential candidate wear-resistant coatings

Several coatings are being investigated for their potential to reduce wear in joint implants. The following section will examine the published research, categorized into six groups according to coating composition: diamond-like carbon, silicon nitride, chromium nitride, zirconium based, titanium based and tantalum based. A summary of the properties can be found in Table 3 (hardness, Young's modulus and adhesion) and Table 1 in supplementary information (surface roughness and wear properties). The properties vary, with hardness ranging from 8 to 44 ​GPa and Young's modulus from 100 to 466 ​GPa. The tribological properties such as wear rates were obtained using different set-ups, with differences in contact pressure and counter surface, leading to large inherent variation between samples, making results difficult to compare.

**Table 3:** Hardness and Young's modulus, as obtained with nanoindentation, and adhesion test values for the reviewed coatings.

Coating (substrate)	Deposition technique	H [GPa]	E [GPa]	Adhesion from scratch Test [N]^a^	Reference
DLC (CoCr)	Unbalanced MS	13	100		Guo et al., 2015 [129]
DLC (CoCr)	PECVD	24			Thorwarth et al., 2010 [130]
DLC (cemented carbide)	Enhanced cathodic arc MS	16.7	166		Wang et al., 2015 [131]
F-FLC (Si)	PECVD	16.41	132.65		Wang et al., 2020 [132]
SiN_x_ (CoCr)	HiPIMS	12–26	173–293		Skjöldebrand et al., 2017 [47]
SiN_x_ and SiN_x_C_y_ (CoCr and Si)	HiPIMS	18	200		Pettersson et al., 2013 [49]
SiN_x_ (CoCr and Si)	RF MS	18–24		0–7	Olofsson et al., 2012 [53]
SiNO and F:SiCN (CoCr)	Unbalanced MS	15	236		Shi et al., 2012 [50]
SiN_x_ (CoCr)	HiPIMS			14–88	Filho et al., 2019 [52]
SiN_x_, SiCN, SiCrN and SiNbN (CoCr)	HiPIMS	13–25	148–286		Filho et al., 2019 [51]
SiN_x_ (CoCr)	HiPIMS				Filho et al., 2020 [46]
TiCN (Ti6Al4V)	Cathodic arc deposition	8–10			Sáenz de Viteri et al., 2015 [41]
TiN (CoCr)	MS	21–23		45–70	Gallegos-Cantú et al., 2015 [42]
Multilayered TiN/CrN (CoCr)	MS	8.0–13.5		50–70	Gallegos-Cantú et al., 2015 [42]
TiN (cemented carbide)	Enhanced cathodic arc MS	23.6	397		Wang et al., 2015 [131]
TiAlN (cemented carbide)	Enhanced cathodic arc MS	27.3	466		Wang et al., 2015 [131]
Multilayered TiAlN (Ti6Al4V)	Closed field unbalanced magnetron sputter ion plating	18.8–44.1	302.6–516.5	17-7-47.7	Yi et al., 2016 [54]
TiN (Ti or Ti6Al4V)	Laser nitriding	997-1099 HV			Chan et al., 2017 [43]
Nitrated TNZT	Laser nitriding	14	171 (Er)		Chan et al., 2016 [133]
TiC (steel)	PECVD	829-1500 HV		40–70	Vitu et al., 2008 [134]
CrN/NbN (CoCr)	HiPIMS	34	447	50–100	Hovsepian et al., 2016
CrN (cemented carbide)	Enhanced cathodic arc MS	17.9	422		Wang et al., 2015 [131]
CrN (CoCr)	Plasma nitriding	12–19			Liu et al., 2013 [135]
CrCN (CoCr)	Plasma carbonitriding	16–18			Liu et al., 2013 [135]
CrN and Cr2N (CoCr)	Plasma nitriding	660-900 HV			Wang et al., 2010 [136]
CrN/NbN (Stainless steel 304)	MS	28	390	0.02	Huang et al., 2017 [45]
CrAlTiN (Stainless steel 304)	MS	33	450	30.4	Huang et al., 2017 [45]
Multilayer TaC and Ta_2_C (CoCr)	Thermal treatment in molten salts	24–37	250–316	LC3: 11-48	Balagna et al., 2012 [36]
TaN(CoCr)	RF sputtering	15–28	255–319		Corona-Gomez 2021

a L_C2_ according to ISO 20502 unless otherwise indicated.

### 5.1 Diamond-like carbon and nano-crystalline diamond

The term diamond-like carbon (DLC) covers a range of hard carbon-based materials with a wide range of properties such as hardness and wear resistance. The variety of structures and consequently properties for carbon is explained by its ability to exist in three hybridizations (sp^1^, sp^2^ and sp^3^). DLC coatings have a significant fraction of sp^3^ bonds [137]. However, it is important to keep in mind that DLC coatings do not consist solely of amorphous carbon (a-C) but also hydrogenated amorphous carbon (a-C:H) alloys and the structure of the coating will largely depend on the deposition method. For example sputtered coatings typically can extend from sp^2^ to sp^3^ and plasma-enhanced CVD produced coatings have a higher fraction of hydrogenated carbons [137].

DLC coatings have been considered for joint implants because of the promise of a chemically inert, hard, wear resistant surface and significant research has been conducted on DLC-coated implants industrially; e.g. in 2001 the company Implant Design AG put forward a DLC-coated knee implant but had to withdraw it the same year after it was banned by the Swiss Federal Office of Public Health (SFOPH). The implants failed due to excessive wear caused by partial delamination of the coating, which led to early revisions [128,138]. Since then research has focused on understanding the mechanism behind the failures as well as improving the coating adhesion. Falub et al. observed an interlayer measuring approximately 5 ​nm in thickness that occurred gradually between the CoCr implant and DLC coating. This interlayer consisted mainly of carbides with an overall stoichiometry close to Me_2_C and delamination is believed to be caused by the instability of Co carbides in this interlayer (Fig. 1) [139]. The mechanism is believed to be stress-corrosion cracking, i.e. delayed failure due to environmentally induced crack propagation [140].

Attempts to improve the adhesion by using interlayers was made by Thorwarth et al. [37] in a study whereby tantalum (Ta) was used as an interlayer for a DLC coating. The results were promising, with little noticeable wear for DLC coatings with Ta interlayers in the case of low concentrations of oxygen impurities. However, it was hypothesised that oxygen contamination would lead to an increased occurrence of the β-phase in the Ta interlayer, which could lead to mechanical failure due to its brittleness. Wang et al. on the other hand proposed the introduction of a fullerene-like structure and incorporating fluoride (F-FLC) to obtain long-term stability in *in vitro* environments [132]. The results revealed coatings with lower coefficient of friction and wear compared with DLC as well as promising *in vitro* results with e.g. cell adhesion of rabbit bone marrow mesenchymal stem cells. In addition Cr has been used to in an attempt to improve adhesion and these coatings exhibited improved corrosion resistance compared with CoCr [141].

The closely related nanocrystalline diamond coatings (NCD) consist of nano-sized diamond crystals in the range of 3–15 ​nm with a large fraction of amorphous carbon at the grain boundaries [142]. These coatings have similar wear properties to DLC coatings and have been shown to reduce the wear compared to uncoated Ti6Al4V components [143]. However, as previously mentioned CoCr is the common choice for articulating surfaces due to its superior wear properties, and a comparison to this material is lacking. Cell studies using primary human bone marrow cells have shown cell attachment, spreading and proliferation on the coatings, i.e. non-cytotoxicity. The available biocompatibility studies however seem to focus on osteoblasts, which may be more suitable for coatings aimed at bone ingrowth [[144], [145], [146]].

After investigating and addressing the risk of delamination in DLC coatings the use of interlayers could make them a viable option for joint implants as observed from the low wear rates (Table 1 in supplementary information section) [38,129,147].

### 5.2 Silicon nitride

Bulk silicon nitride, Si_3_N_4_, and related materials have been used in applications such as combustion engines due to high temperature and wear resistance [148], and components made of Si_3_N_4_ have been used in several applications subjected to wear, e.g. bearings [[149], [150], [151], [152]]. It is also used in biomedical applications such as spinal implants [153].

The main advantage of silicon nitride, SiN_x_, as a coating for joint implants has been shown to be, not only its ability to reduce wear, but also to minimize the adverse immune response to released ions and particles. SiN_x_ dissolves in aqueous solutions into only biocompatible elements [154], which could mean that the generated wear particles would dissolve in the body without triggering the immune system response that would eventually lead to bone resorption. It should be noted however that ammonia is formed during the dissolution of SiN_x_, which may result in an elevated pH. However, this has been found to be beneficial in terms of antibacterial properties [155,156]. Further, there needs to be a compromise between the dissolution of the wear particles and the requirement of a coating that provides sufficient performance for the intended period of use i.e. significantly greater than 20–25 years.

SiN_x_ coatings have been manufactured both through PVD and CVD methods with a wide range of hardness (12–24 ​GPa) [47,49,50,53] and Young's moduli (173–293 ​GPa) [47,49,50]. Specific wear rate measurements through pin-on-disc setups have found reduced wear compared to CoCr [49,53]. The generated wear debris was investigated in a study where a ball-on-disc set-up was used to produce the same, which was found to lie in the range of 0.01–0.05 ​μm [157]. It was noticed that the debris agglomerated and that these agglomerates were in the range of 0.15–1.96 ​μm, while the individual particles measured between 0.01 and 0.05 ​μm in size. It was also found that the pH increased from 7.45 to around 8 after 20 days, likely due to the formation of ammonia [158,159], as mentioned above.

Biocompatibility studies have mainly been focused on simulated wear particles, often commercially available alternatives. The particles, both micron-scale and nanoscale, did not give rise to any significant release of proinflammatory cytokines in a study with primary human peripheral blood mononuclear cells [160]. Bulk Si_3_N_4_ has also been found to show no cytotoxic effects [161,162] but instead antiviral properties [163].

**Fig. 2:**
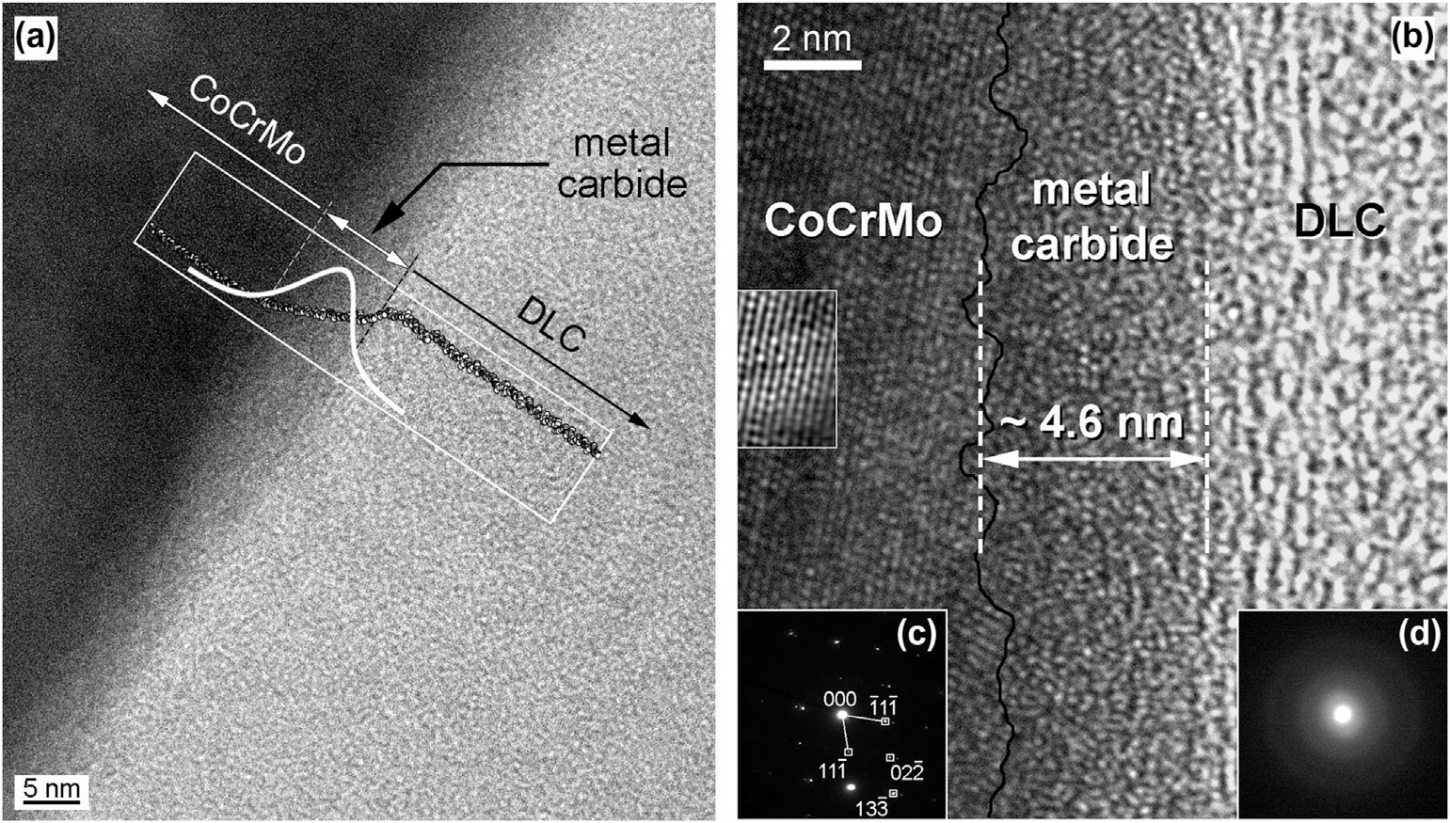
**Transmission electron microscopy (TEM) investigations of the interface of a DLC coating on a CoCr substrate. Both the TEM image (a), high resolution TEM image (b) reveal the presence of a metal carbide, which could lead to delamination when exposed to the environment of the body** [139]**. Reprinted by permission from Acta Materialia, Elsevier.**

The aforementioned dissolution behaviour of the coatings was investigated in simulated body fluid (25 ​vol% foetal bovine serum diluted in phosphate buffer saline solution) for up to 60 days (Fig. 2). The coatings successfully reduced the metal ion release by two orders of magnitude, as compared to uncoated CoCr references. The dissolution rates of the coatings were lower or comparable to the CoCr (0.2–1.2 ​nm/day for SiN_x_ coatings compared to 0.7–1.2 ​nm/day for CoCr) [48]. It should be noted however, that the dissolution rate will depend on factors such as composition and density of the coating - an increased nitrogen content has e.g. found to yield lower dissolution rates [48]. Recent studies have also found that alloying the SiN coating with Fe and C or Cr and Nb could give reduced dissolution rates [164].

### 5.3 Chromium nitride

Similar to other ceramic coatings CrN coatings have been shown to increase hardness and reduce wear compared to CoCr [44,165], as well as reducing the release of metal ions. The added advantage of CrN coatings is the possibility to achieve a CrN surface layer through e.g. plasma nitriding. Because of its potential for strong adhesion CrN is also sometimes used as an interlayer between the substrate and a top coating [46,52].

In a study comparing the performance of CrN with TiN, TiAlN and DLC, CrN was found to exhibit superior corrosion and wear resistance [131]. CrN coatings have been subject to additional investigation whereby coated CoCr femoral heads were paired with PE cups and tested in a hip simulator for 5 million cycles. The CrN coatings were shown to produce similar amounts of PE wear under standard conditions compared to adverse conditions (9.5 mm^3^/mc and 12 mm^3^/mc for standard and adverse conditions – for the latter alumina particles were introduced to simulate third body abrasive wear), while the uncoated CoCr head showed a large increase in wear for the adverse conditions (9.2 mm^3^/mc and 469 mm^3^/mc under standard and adverse conditions, respectively) [166]. Another study comparing TiN, CrN, CrCN and DLC coatings in a hip simulator showed a 36-fold reduction in wear rate for self-mating CrN and CrCN coatings compared with uncoated CoCrMo MoM implants. In addition, the ion release was dramatically reduced [165].

Another method of creating a CrN rich surface is through incorporation of nitrogen into the surface of CoCr using reactive plasma. This was reported by Wang et al. [136], who exposed CoCr substrates to a plasma of NH_3_ at a constant pressure of 500 ​Pa for 9 ​h. The formation of a CrN and Cr_2_N layer was confirmed by X-ray diffraction (XRD), and evaluation showed that the nitrided surface was harder and had lower wear rates compared to the untreated surface when in contact with a cemented carbide (WC/Co) ball during ball-on-disc wear tests. By using different plasma gases it is possible to obtain both nitrided and e.g. carbonitrided surface layers [135]. This was investigated by Liu et al. [135], who found both plasma nitriding and plasma carbonitriding of CoCr increased the hardness and wear resistance as compared to untreated CoCr. In addition, corrosion resistance was improved for both treatments, and the carbonitrided surface showed a better corrosion resistance compared to the nitrided surface.

Reported wear rate values of CrN based coatings have been consistently low (1∙10^−6^ to 8.8∙10^−7^ mm^3^/Nm) [44,167] and adhesion tests have given high critical load values (L_C2_) - up to 50 ​N for CrN/NbN deposited on CoCr substrates [44,45]. Overall the low wear rates and contingency of good adhesion make CrN-based coatings potentially suitable for joint implants.

### 5.4 Zirconia and yttria-stabilized zirconia

Zirconia and yttria-stabilized zirconia have been investigated as potential candidate coatings for orthopaedic implants because of their ability to reduce wear. The number of studies available on these materials for this application are still limited but the results are comparable to other investigated coatings [168,169]. These coatings were deposited onto a substrate such as titanium and should not be confused with the surface treated Oxinium implants.

The wear properties of yttria-stabilized zirconium dioxide (YSZ)-coated titanium balls paired with UHMWPE have been evaluated in a ball-on-disc set-up using different lubricants. The coatings were found to decrease the wear of the PE under dry conditions and when lubricated with a NaCl solution. However, when lubricated with a serum solution i.e. under conditions more closely emulating those of a natural joint, the results were similar to those obtained for the uncoated Ti spheres [169] (3–13∙10^−4^ mm^3^/Nm for dry conditions and 3–8∙10^−4^ mm^3^/Nm for serum solution as a lubricant; the root mean square surface roughness of the coatings ranged from 28 ​± ​1 ​nm to 60 ​± ​5 ​nm). The coatings did not exhibit any cytotoxicity when tested with mesenchymal stem cells and pre-osteoblast cell lines [168], indicating that they are biocompatible and could be useful as coatings for orthopaedic implants.

One way of achieving a surface oxide layer is through thermal oxidation, this was conducted by Luo et al. who oxidized a ZrNb alloy. An increased treatment temperature (of 700 ​°C compared to 500 ​°C) improved both the hardness and wear resistance [170].

The hydrolytic long-term stability of zirconia-based material remains a concern however [171,172], and would have to be thoroughly investigated before being an option for biomedical implants.

### 5.5 Alloyed and structured titanium nitride and carbide

While titanium nitride has successfully made it to the market (Implantcast, Cellumed, Link medical technology and Link orthopaedics) there is still ongoing research aimed to improving these coatings. One such strategy is to alloy the TiN coating with one or more elements and another is using a multi-layer structure. These strategies have the potential to further reduce the wear and/or improve adhesion.

Although alloying TiN with elements such as aluminium has been found to increase the hardness and Young's modulus, and decrease the wear rate compared to Ti6Al4V, however it should again be noted that CoCr is more commonly used in articulating surfaces and should be the material used for comparison [54]. Other investigated alloying elements include niobium and carbon, which have been shown to be non-cytotoxic [39]. Titanium carbide has also been shown to reduce wear compared to uncoated Ti6Al4V when tested in a reciprocating pin-on-disc set-up [173].

Another strategy for the improvement of wear properties is to deposit multilayers. The mechanical and tribological properties of such a coating (TiN/CrN) has been investigated and compared to a TiN monolayer. The multilayer structures reduced the friction coefficient, however, the wear rate was not reported [42].

Another option is to create a ceramic surface layer, by e.g. heat-accelerated diffusion. There are several techniques available for alloying the surface of a material by exposure to elevated temperatures in a controlled environment. Another example is the powder immersion reaction assisted coating (PIRAC). Yet another process whereby nitrogen is incorporated into the surface is laser nitriding, where the substrate is irradiated with a laser in a chamber with nitrogen gas. The laser-illuminated area of the surface will melt and create a plasma above it. Subsequently the nitrogen, which is now ionized, will be absorbed by the melted surface. All methods result in increased hardness as well as increased wear resistance compared with Ti6Al4V. The PIRAC method and laser melting did however yield an increased surface roughness compared to an untreated reference [43,133,[174], [175], [176]].

While TiN based coatings offer a possibility to improve the wear properties of Ti6Al4V, they are in many of the published current studies compared only to uncoated Ti6Al4V, which makes it challenging to assess the efficacy of these proposed coatings.

### 5.6 Tantalum carbide, tantalum oxide and tantalum nitride

Porous tantalum is used in orthopaedic applications such as cranioplasty plates and hip implant fixation due to its osseintegrative properties [177]. Proven to be non-cytotoxic, tantalum-rich coatings, such as TaN and TaO, have been proposed for application on the articulating surfaces of joint implants due to their corrosion resistance, promising results *in vitro* [177] and favourable mechanical properties.

Tantalum carbide coatings deposited on CoCr by thermal treatment in molten salts have been investigated with regards to their adhesion, mechanical and tribological properties [36,178]. The coatings were found to have a TaC or Ta_2_C-TaC structure depending on the carbon content, manufacturing process of the CoCr substrate and temperature during coating growth. In addition, the coatings were deposited in a multilayer structure with layers comprising different carbon contents. The thickness of the coatings varied depending on structure and ranged from 300 to 1000 ​nm. The structure of the coating proved important for adhesion, where a multilayer structure (TaC-Ta_2_C-Ta) yielded higher critical loads during scratch tests (delamination at 30 ​N compared with delamination at 14 ​N for a single layer TaC) [36]. When evaluating hardness and Young's modulus by nanoindentation (with a maximum load of 10 ​mN), the hardness was significantly increased (27 ​GPa for a multi-layer structure and 23 ​GPa for a single layer coating compared to 12 ​GPa for the uncoated substrate) [178]. The wear volume obtained in a pin-on-disc set-up with 25 ​vol% bovine serum diluted in distilled water as lubricant was similar for the different multi-layer structures. The wear was reduced compared to the uncoated substrate, however there were no discernible differences between coatings in terms of wear performance. Noteworthy is that during wear testing, third body abrasion was the most prominent wear mechanism due to pull-out of carbides that acted as third body abrasive particles.

Investigation of the corrosion and wear resistance of tantalum oxide (TaO_2_) deposited onto Ti6Al4V has shown improved corrosion properties with an increased corrosion potential, reduced anodic current and reduced Ti ion release (I_corr_ of 6.770∙10^−8^ A/cm^2^ for TaO compared to an I_corr_ of 2.560∙10^−7^ A/cm^2^ for Ti6Al4V). The wear volume of the coated samples was reduced compared to Ti6Al4V (2.22 mm^3^/Nm compared to 7.78 mm^3^/Nm for uncoated Ti6Al4V) [179]. Again, a limitation of the study was the comparison only to Ti6Al4V. Another study where TaN was deposited on CoCr by RF sputtering the coatings were shown to have comparable or lower wear rates of a PE counter surface to uncoated CoCr [180].

In summary, tantalum carbide and oxide coatings have been shown to be biocompatible, but their wear performance in the application requires further investigations.

### 5.7 Alumina based coatings

In addition to the previously discussed coatings alumina based coatings have been proposed as an option. These coatings include monolithic micron alumina (IDA), micron alumina yttria-stabilized zirconia (YSZ) composite coating (IDAZ), and nanostructured alumina titania/YSZ (IDZAT) deposited on Ti-6Al-4V alloy and have been shown to have better wear and corrosion compared to Ti6Al4V [181].

## 6 Discussion

This review has provided a comprehensive overview of both commercially available coated implants, as well as coatings currently being researched for potential application in joint implants. The coated implants currently available in the market are ZrO_2_ coated Zr (Smith and Nephew), TiN coated CoCr or Ti6Al4V (Implantcast), Link orthopaedic), TiNbN coated CoCr (OHST medical technology) and TiN coated Ti6Al4V (Endotec). Reported follow up studies as well as case studies of retrieved implants have revealed revision rates comparable to traditional CoCr implants [104,105,[124], [125], [126]]. The potential coating materials currently being investigated include SiN_x_, CrN, TaO, TaC, DLC, TiN, TiCN, ZrO_2_ and ZrN. Looking at total knee replacements the use of coatings is more widespread and the retrieval data reveals the coated implants to be comparable to CoCr but there is not enough evidence to support a lower amount of osteolysis-caused failures [80,113,[116], [117], [118], [119], [120], [121], [122], [123], [124], [125]].

**Fig. 3:**
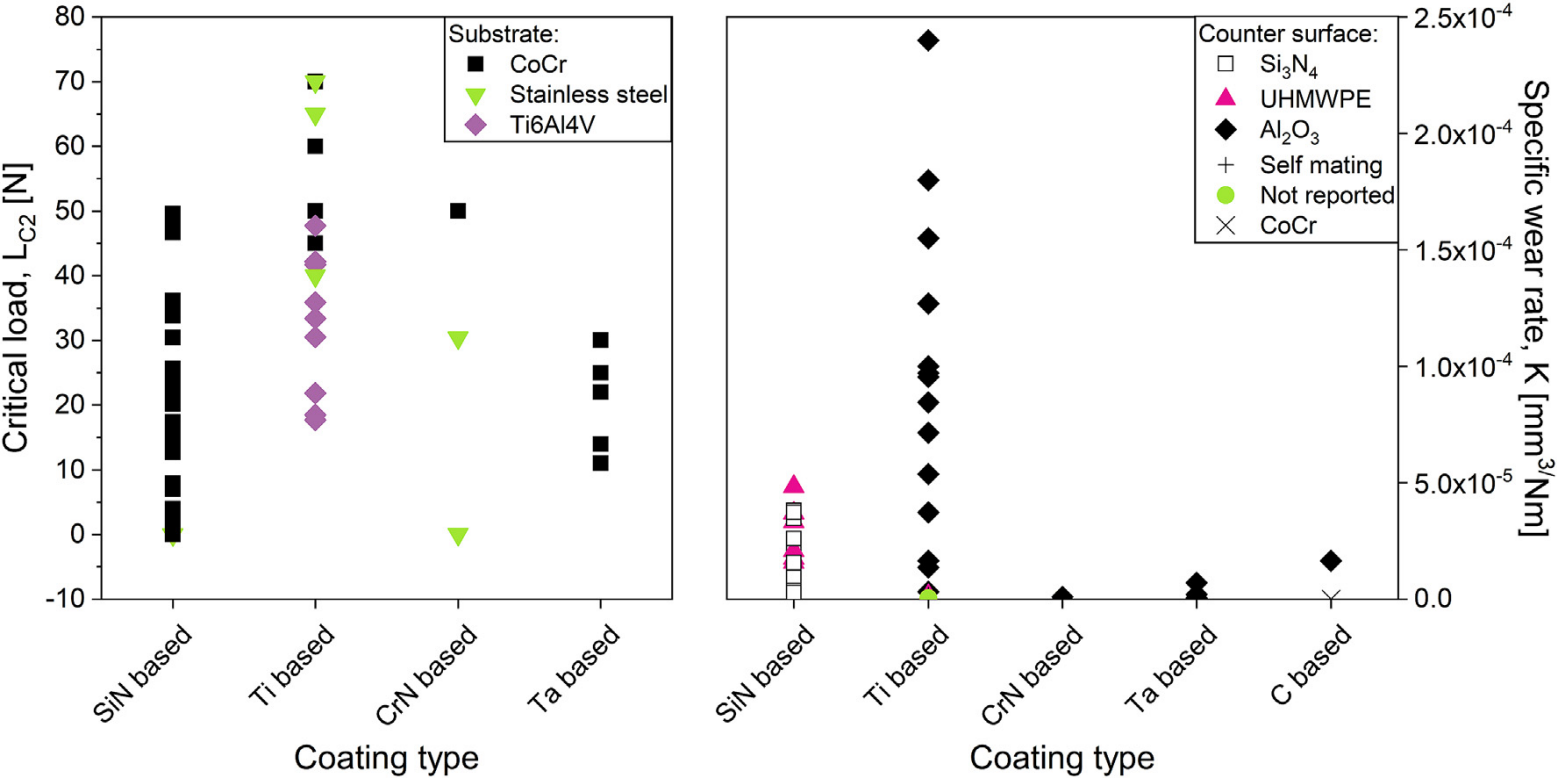
A comparison of the LC_2_ values [36,45,[51], [52], [53], [54],^134^] (a) and specific wear rates from ball/pin-on-disc evaluations [46,49,[51], [52], [53], [54],^129^,^131^,[134], [134],^173^,^179^,^183^] (b) found for coatings currently being researched. The reported values are divided into groups based on the main constituents of the coating.

When comparing the specific wear rates of coatings currently being researched (Fig. 3b) it is evident that all coating types (silicon nitride based, titanium based, chromium nitride based, tantalum based and DLC) have the potential for low wear rates. However, it is difficult to distinguish between them since i) different set-ups have been used to assess the wear performance, and ii) different counter surfaces have been used in the tests, i.e. hard-on-hard (e.g. alumina) or hard-on-soft (a polymer). The available standards cover procedures for both types of material combinations, including suitable testing conditions. However, even when the testing is performed in accordance with the standards it might not reflect the full range of loading scenarios the implant will be exposed to. To better predict clinical outcome the current standardized approaches require further development. Important considerations for such an approach are of course the choice of material combinations, i.e. hard-on-hard versus hard-on-soft as well as the effect of activities other than steady state gait. Common activities such as standing up from a chair and climbing stairs put a higher demand on the implant and will affect the implant performance.

The previous introduction to and subsequent removal from the market of DLC coated implants illustrates the need for more rigorous testing before clinical use. DLC coated implants were removed from the market after the revelation that exposure to synovial fluid over time led to a delamination of the coatings due to the instability of metal carbides in the interlayer, which in turn caused early revisions. Scratch and Rockwell tests typically evaluate the adhesion of as-deposited coatings, but typically do not consider corrosion or changes to the coating over time. To better predict the adhesion of the coating, the scratch or Rockwell tests can e.g. be performed on coatings exposed to liquid at different soaking times [52]. In this review, DLC coatings were included as materials that are not currently in the market but show potential for implant applications. Having been introduced to the market and suffered from early implant revisions, DLC coatings are often not considered an option. However, the mechanisms causing the delamination have since been investigated and are now better understood. By using interlayers, DLC coatings could be stable long-term, reduce wear and potentially increase implant longevity.

In summary, several of the investigated coatings show potential because of their ability to reduce wear and ion release. Different coatings carry different advantages, e.g. CrN on CoCr and TiN on Ti6Al4V can give enhanced adhesion. One material that has the potential to provide additional biological advantages is silicon nitride, which has demonstrated antibacterial and antiviral properties.

Revision and follow up studies of commercially available coatings have found revision rates to be comparable to, but not better than, conventional CoCr implants. This could be due to low revision rates or not long enough follow-up studies (Table 2) [55,80,96,[104], [105], [106], [107], [108], [109], [110], [111], [112], [113], [114], [115], [116], [117], [118], [119], [120], [121], [122], [123], [124], [125], [126], [127], [128]]. The signs of wear and damage of the PE countersurface has been found to be lower for oxidized zirconium knee implants compared with CoCr [120], which is promising as it speaks to the potential for reduced generation of PE debris. Since this debris is believed to be a major cause of revision its reduction could lead to a longer implant lifetime. Assuming the coating adheres well to the substrate it is possible to have a biocompatible surface that generates low amounts of wear debris and ion release, which would be ideal for articulating surfaces.

Whilst much of the coating work has focussed on the application of coatings to bearing surfaces to reduce wear and corrosion, there is now also a growing interest in translating such technologies to other interfaces, such as hip modular-tapers, where fretting-corrosion processes may dominate. The ability to develop novel multi-material systems also paves the way for a new generation of multi-functional coating technologies to combat the aforementioned issues as well as the emerging grand challenges within the area of orthopaedics (e.g. infection and treatment of metastatic cancers).

## 7 Conclusions

In conclusion,

• Coated implants are available in the marked. These implants include TiN, TiNbN, ZrN coatings and surface treated Zr resulting in an oxidized surface layer.
• Several candidate coating materials such as carbon-based, silicon nitride, chromium nitride, Ti-based, Zr-based, Ta-based and alumina-based are being researched.
• Coated implants exhibit comparable survival rates to uncoated implants, however the basis for assessment is limited due to generally low revision rates and short follow-up times.
• Coatings could be relevant for other surfaces such as modular interfaces.

## Data availability

The data in this review consists of information found in published research papers.

## Declaration of competing interest

The authors declare that they have no known competing financial interests or personal relationships that could have appeared to influence the work reported in this paper.
