# Supplementary material for: Current status and future potential of wear-resistant coatings and articulating surfaces for hip and knee implants
Source: Mater Today Bio. 2022 Apr 30;15:100270. doi: 10.1016/j.mtbio.2022.100270 (PMC9118168; doi:10.1016/j.mtbio.2022.100270)
Supplement: Multimedia component 1 [file mmc1.docx]

Supplementary information – Important properties of wear resistant coatings and surface treatments for the articulating surfaces of hip and knee joint implants

As discussed in the main text there are numerous properties that have to be evaluated for a coating to be introduced to the market. These properties and their evaluation are summarized below.

# Surface roughness

Articulating surfaces generally require a low roughness in order to minimize wear. The maximum average surface roughness of joint implants is specified in ISO 7206-2:2011/Amd1:2016 [1], which states that it should not exceed 50 nm for metals or 20 nm for ceramic materials. While coatings are not included within this standard, they would generally belong to the ceramic class of materials.

The definitions and parameters for use and measurement are defined in the ISO standards ISO 4287 [2] and ISO 4288 [3] for stylus techniques, and in ISO 25178-604:2013 [4] for optical techniques. Besides the average surface roughness, R_a_, other parameters such as the distance between peak and valley, R_z,_ and maximum height, R_t,_ may be of particular relevance to coatings, as they may give an indication of the size and relative quantity of defects.

# Hardness and elastic modulus

The mechanical properties of bearing surfaces, commonly reported as hardness (H) and elastic modulus (E), naturally have a significant effect on an implant’s wear resistance [5]–[7]. A coating with a high hardness is likely to be more resistant to abrasive wear, as is illustrated by the linear wear equation (often referred to as Archard’s equation)

$$V=\frac{kLS}{H}$$

where V is the wear volume, k the wear coefficient, L the load and S the sliding distance. The relevance of Archard’s equation is limited since it assumes that the wear is purely caused by mechanical factors. It has also been suggested that measuring the elastic strain to failure is more suitable as an indicator of wear performance than hardness alone [5]. The elastic strain to failure is related to the H/E ratio and indicates that it is desirable to pair a high hardness with a low elastic modulus. These properties are typically evaluated for coatings through nanoindentation, as it is an established method that allows for shallow measurement whereby influence from the substrate can be avoided. The coating is indented with a tip of known geometry and material properties (most commonly a modified Berkovich tip) to either a maximum depth or load. The most commonly used method for calculating hardness and Young’s modulus was developed by Oliver and Pharr [8], [9]. They propose using the normal force F_N_ and penetration depth p_d_ to calculate hardness according to

$$H=\frac{F_{max}}{A}$$

where H is the hardness, F_max_ the peak applied load and A the projected area at peak load. The stiffness is calculated during initial unloading as a function of the F_N_ and p_d_, which in turn can be used to calculate reduced Young’s modulus (Figure 1). If Poisson’s ratio is known, or can be estimated, it can be used to calculate the Young’s modulus [8], [9].

$$\frac{1}{E_{r}}=\frac{2}{S}\sqrt{\frac{A}{\pi}}=\frac{(1-v^{2})}{E}+\frac{(1-v_{i}^{2})}{E_{i}}$$

where E and ν are Young’s modulus and Poisson’s ratio of the sample and E_i_ and ν_i_ are Young’s modulus and Poisson’s ratio of the indenter.


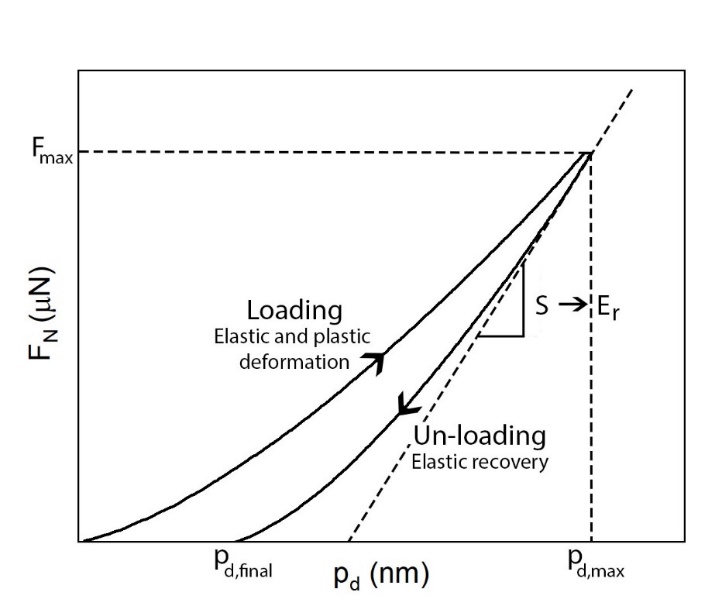


Figure 1. An example of an indentation curve with stiffness, S, maximum load, F_max_, and final, p_d,final_ and maximum depth p_d,max_ marked. During loading the material is deformed, both plastically and elastically, and during un-loading the elastic deformation is recovered.

Nanoindentation should be conducted in a manner where the substrate does not influence the measured hardness or Young’s modulus and typically the indentation depth should be less than 1/10 of the coating thickness. For large discrepancies in hardness between the coating and the substrate, e.g. for a hard coating on a soft substrate, the indentation depth may have to be less than 10% [10].

The procedure for measuring and analysing the hardness and elastic modulus of materials is described in ASTM E2546 – 07 [11] and ISO 14577-1:2015 [12] whilst ISO 14577-4:2016 [13] is specific to the determination of these properties for both metallic and non-metallic coatings.

Target mechanical properties for a coating depend on the counter surface as well as the substrate: a high hardness, while usually beneficial for wear resistance, needs to be balanced with the need for good adhesion, since a large difference in mechanical properties between substrate and coating is likely to have a negative effect on the coating adhesion [14], [15]. Commercially available coatings for joint implants have been reported to have a hardness between 12-28 GPa [16].

# Adhesion

Adhesion describes how well the coating adheres to the substrate, which is critical to coating performance. This is defined as the work (or force) needed to remove a coating from its substrate [17], [18]. However, as this cannot be measured directly, except in very limited circumstances, use is made of various engineering coating adhesion test methods to obtain a quantitative, reproducible adhesion measurement which can be related to the functional performance of the coating [17]. It is crucial to keep in mind that the practical adhesion is influenced by several factors, such as the coating’s residual stress and thickness, possibility for plastic deformation of the coating and/or substrate, and may therefore yield different results depending on the evaluation method. There are a variety of methods available, but the most commonly used for the studies covered in this review are the scratch test and Rockwell indentation test, detailed below.

## Scratch test

The method uses scratching of a coated surface with a diamond stylus of known geometry and under a range of loads to produce adhesive failure of the coating. Scratches may be generated in one of two ways; either multiple scratches using different constant loads or a single scratch using a progressively increasing load. The progressive load scratch test gives a rapid assessment and is often used in research, coating development, and for quality assessment, whilst the constant load scratch method takes longer but provides a greater statistical confidence. To assess the load at which a coating fails, optical evaluation is used to determine where and how this occurs (Figure 2). Friction force and acoustical emission is often also recorded to identify and characterize the failure.


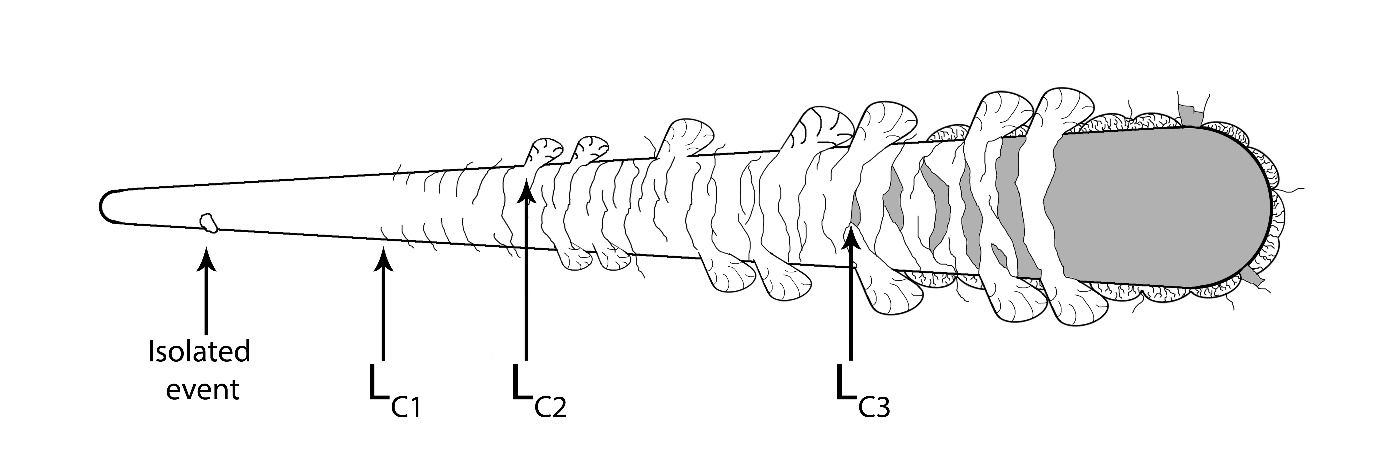


Figure 2. Schematic image of a scratch produced using a progressively increasing load, figure adapted from ISO 20502. The three main categories of failure and their associated critical loads (L_c_) according to ISO 20502 are identified on the image as cracks (L_C1_), chipping, (L_C2_) and exposed substrate in the track, (L_C3_). Ignoring isolated events is common procedure.

The standard ISO 20502 [19] explains the method and gives recommendations for loading rate (100 N/min) and lateral velocity (10 mm/min). The standard is however not specific to coatings for joint implants. There are several different failure modes, L_C_, which can be determined optically. ISO 20502 recommends using a magnification between 100x and 500x and suggests three standardized categories of failure mode: L_C1_ (initial appearance of cracks); L_C2_ (initial coating flake off at the scratch edges); and L_C3_ (initial exposure of substrate in the center of the scratch). The magnification can be import as it is possible to detect smaller failures with a higher magnification.

It should be noted however that the results obtained are dependent on a multitude of factors, including substrate composition, hardness and roughness, coating hardness, thickness, roughness and internal stress, as well as the testing parameters used and the calibration of the instrument. Hence comparison of scratch data is only meaningful for identical coating-substrate combinations tested using the same testing conditions with properly calibrated instruments. In addition, a high L_C2_ values does not necessarily correspond to low wear rates.

## Rockwell indentation

As well as indenting a surface with a conical tip to determine hardness, the Rockwell indentation technique can also be used to assess the adhesion of a coating. The coated surface is indented with a diamond tip with a 200 µm tip radius and 120˚ apex angle. A load of 150 kgf, 100 kgf or 60 kgf is applied depending on substrate material and hardness (150 kgf for metals with hardness higher than 54 HRC, 100 kgf for metals with hardness lower than 54 HRC and 60 kgf for all other substrates e.g. ceramics) [20]. The indent is then evaluated optically to determine the failure mode. According to ISO 26443 [20], failure can be categorised into four groups (0-3), as shown in Figure 3. Another classification of failures has been specified in the VDI standard (VDI3198), where the scale ranges from light cracking (HF1) to complete delamination (HF6). Whilst this standard was withdrawn in 2013 [21], it is still sometimes employed. Coatings are expected to lie within classes ISO 0-1 [20] or HF1, as reported for coatings in clinical use [16], [22]. It should be noted that VDI3198 standard has more requirements regarding coating thickness and substrate hardness while ISO 26443 is more generally applicable. In general scratch testing is more widely used, however Rockwell indentation is useful for samples with a more complicated geometry where a scratch is not possible due to complicated or curved surfaces. It is also less sensitive to differences in thickness compared with scratch testing, which could make it possible to compare samples.


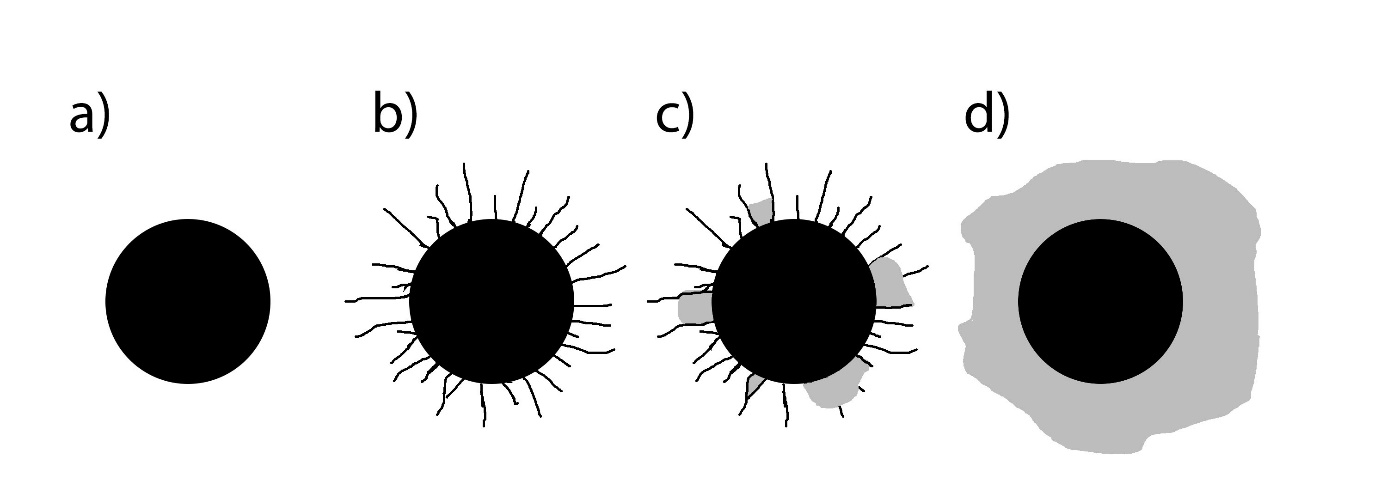


Figure 3. The four categories of failure mode according to ISO 26443 (figure adapted from same standard), showing (a) Class 0; no sign of delamination or cracking, (b) Class 1; cracks but no delamination, (c) Class 2; partial delamination (with percentage estimate of delamination in relation to indent surface area) and (d) Class 3; complete delamination.

# Wear and degradation

There are several factors contributing to the degradation of a coating *in vivo*, the most prominent being corrosion and wear and it is typically not possible to isolate one from the other.

## Corrosion

Degradation through dissolution, or corrosion, may cause ion release *in vivo*. In the human body, biological reactions may also take place that can increase the degradation rate, for example the pH may decrease during inflammation [23]. A coating’s dissolution or corrosion rate can be characterized in several ways. Studies tend to adopt immersion techniques where coatded samples are immersed in simulated biological fluids. In-situ electrochemical (open circuit potential (OCP) [24] and linear polarisation scans (LPR) [25]), gravimetric mass loss [26], ion release measurements (ICP-MS/AAS) and surface metrology have been used to quantify coating loss owing to corrosion. Degradation rates may also be investigated through *in vivo* implantation and through revision explant analyses [27]–[29].

In-situ electrochemical techniques provide a convenient method to assess the corrosion rate in real time. However, these are not without their limitations, particularly in the case of localised corrosion, and complementary aforementioned analysis is often required. A coating is expected to last at least 15 years based on an average age of implantation of approximately 65-70 years [30]–[38] for hip and knee replacements and a life expectancy of approximately 81 years (based on the average life expectancy in the countries from which joint registers were obtained i.e. United States, Canada Australia, New Zealand, the U.K, Norway, Denmark and Sweden) [39]. It should be noted that while the average age at implantation is 65-70 a lot of the patients are younger than this. As the addition of a coatings is expected to prolong implant lifespan it is reasonable to expect an implant lifetime of 20-25 years. Hence the dissolution rate needs to be much lower than this in the final application, especially considering wear might exacerbate this. In addition the dissolution may be accelerated by the presence of coating defects or a columnar structure [23].

## Wear rate

The main focus of evaluation is often wear and the generated wear particles, which are believed to have a significant effect on an implant’s longevity [40], [41]. It is therefore important to evaluate both the wear resistance of the coating (supported on the same substrate material, in the same condition to be used for the final application) and that of the counter face, as well as the properties of the wear debris that is produced.

The complexity of the evaluation of wear performance ranges from simple laboratory “2D” measurements, such as pin-on-disc tests, to “3D” simulator wear tests where the articulation and environment of the joint is replicated, to explant evaluation.

There are a wide range of set-ups available to investigate coating wear properties [42]–[46]. The simplest of these use a pin or ball to slide against a flat surface (e.g. disc or plate), either in a reciprocating or revolving pattern. The contact geometry, load, resulting contact pressure, velocity, stroke length, diameter of the wear track and lubricant are typically chosen to simulate conditions in actual joint replacements and the parameters can all be varied during or between tests. The level of wear can be estimated after the test by measuring either the dimensions of the wear scar or mass loss of the sample, and is often reported as volume loss per unit of sliding distance. Procedures are specified in EN 1071-12 [47], ASM F732 [42] for polymeric materials, ISO 20808 [48] for ceramic materials and EN 1071-13 for ceramic coatings [49].

In order to undertake a rapid wear rate assessment, it is common to intensify the conditions by e.g. using a higher contact pressure, although this has the limitation of no longer mimicking the *in vivo* condition and may affect the wear mechanism. The choice of lubricant has been shown to affect the wear rate. Reported lubricants vary from distilled water, saline solutions and serum solutions (most commonly foetal bovine serum). The presence of proteins in the lubricant has a major effect on the wear properties [43], [50], [51] and it is therefore advised to use e.g. a bovine serum as a lubricant as specified in the standards ASTM F1714-96 [43] and ASTM F732 [42] and ISO 14242-1 [44] and ISO 14243-3 [52].

A set-up with a multidirectional movement pattern and a more advanced geometry e.g. ball-cup “hip-type” simulator test can be used to better simulate physiological conditions albeit in terms of a benign walking cycle, as specified in ISO 14242 [44], [46], [53], [54] and ASTM 1714-96 [43]. Similar set-ups exist for other joints e.g. the knee as specified in ISO 14243 [45], [52], elbow[55] and shoulder [56]. Whilst these multidirectional movement tests are more expensive and time-consuming than the simpler set-ups, they are also necessary before clinical trials as they more closely mimic the intended loading conditions. Testing in scenarios that challenge the coating under more extreme activities of daily living can be extremely useful in ascertaining the performance of coated bearing surfaces in adverse conditions [57], [58] for instance, limited edge loading has demonstrated high levels of wear, particularly in resurfacing or other metal on metal joints.

Table 1 shows wear and surface roughness data from a number of published studies on various coating/substrate combinations of interest for orthopedic joint applications and prepared using a variety of coating techniques. From this table it is evident that a variety of different test methods are used with different set-ups, lubricants and counter surfaces, with results being express in different units. This illustrates the difficulties in comparing data from different studies and it is therefore important to always have a reference material when conducting such studies. Relevant reference materials when investigating wear properties are the materials used in articulating surfaces of joint implants, such as CoCr. Other materials such as Ti are not used for bearing surfaces and are therefore typically not suitable reference materials.

An important factor when conducting tribological tests is the lubricant [59]. It is therefore important to select a lubricant that resembles the synovial fluid. One important factor is the presence of proteins which has been found to influence the tribological outcome and even lubrication regime [59], [60].

In conclusion, while wear testing is necessary it is difficult to compare studies. It might be useful to employ simplified set-ups and testing conditions for screening e.g. a range of compositions. However, to have an indication of the performance *in vivo* or in patients it is of the utmost importance to use realistic conditions and always compare with a reference material or implant.

Table 1. Values of surface roughness (Ra) and wear properties of various coating-substrate combinations reported in scientific papers.

| **Coating (substrate)** | **Deposition technique** | **Ra [nm]** | **Wear** | | | | | | **Reference** |
| --- | --- | --- | --- | --- | --- | --- | --- | --- | --- |
|  |  |  | **Method** | **Lubricant  (* indicates proteins)** | **Counter surface** | **µ** | | **Wear rate or mass loss** |  |
| DLC (CoCr) | Closed-field unbalanced magnetron sputtering (PVD) | 10 | Pin-on-disc | * Bovine serum albumin solution (BSA) under different concentrations | CoCr |  | | 1.6∙10^-8^ mm^3^/Nm | Guo et al. 2015 [51] |
| DLC (CoCr) | CVD |  | Hip simulator | * Bovine serum containing solution (30 g/l) | DLC(CoCr) |  | | 3.4∙10^-3^ mm^3^/1000 cycles | Throwarth et al. 2010 [61] |
| DLC (CoCr) | Magnetron sputtering (PVD) |  | Simplified knee simulator | Distilled water | UHMWPE |  | | 0.15 mg/5 Mc | Oñate et al. 2001 [62] |
| DLC (austenitic stainless steel) | CVD |  | Hip joint simulator | Distilled water | UHMWPE |  | | Approx. 10 mm^3^ on coated femoral heads after 6 Mc | Dowling et al. 1997 [63] |
| DLC (cemented carbide) | Enhanced cathodic arc magnetron sputtering (PVD) |  | Ball-on-disc | Simulated body fluid | Al_2_O_3_ | 0.372 | | 1.64∙10^-5^ mm^3^/Nm | Wang et al. 2015 [64] |
| NCD (Si_3_N_4_) |  | 20-30 | Hip joint simulator | * sterilized alpha calf serum (30 g/l) | NCD(Si_3_N_4_) |  | | Femoral head: 0.022±0.009 mm^3^/Mc Acetabular cup: 0.14±0.03 mm^3^/Mc | Maru et al. 2015 [65] |
| SiN_x_ and SiN_x_C_y_ (CoCr and Si) | High power impulse magnetron sputtering (PVD) |  | Ball-on-disc | * 25% fetal bovine serum | Si_3_N_4_ | 0.2 - 0.3 | | 1.3-240∙10^-7^ mm^3^/Nm | Pettersson et al. 2013 [66] |
| SiN_x_ (CoCr and Si) | RF magnetron sputtering (PVD) | 10-250 | Ball-on-disc | * 25% fetal bovine serum | Si_3_N_4_ | 0.13 - 0.31 | | 2.7-3.2∙10^-7^ mm^3^/Nm | Olofsson et al. 2012 [67] |
| SiN_x_ (CoCr) | High power impulse magnetron sputtering (PVD) | 8-33 | Pin-on-disc | * 25% fetal bovine serum | UHMWPE |  | | 0.3-113 mm^3^/MC (PE counter surface) | Filho et al. 2019 [68] |
| SiN_x_, SiCN, SiCrN and SiNbN(CoCr) | High power impulse magnetron sputtering (PVD) | 30-43 | Femoral head-on-disc | * 25% fetal bovine serum | UHMWPE | 0.11-0.18 | | 1.59-4.84∙10^-5^ mm^3^/Nm (PE counter surface) | Filho et al. 2019 [69] |
| SiN_x_ (CoCr) | High power impulse magnetron sputtering (PVD) | 16-33 | Ball-on-disc | * 25% fetal bovine serum | Si_3_N_4_ | 0.35-0.42 | | 5-381∙10^-7^ mm^3^/Nm | Filho et al. 2020 [70] |
| TiN (AISI 316L steel) | Arc evaporation (PVD) | 6-10 | Pin-on-disc | * Bovine serum albumin | UHMWPE | 0.25-0.38 | 1-8∙10^-3^ mm^3^/Nm (PE counter surface) | | Serro et al. 2009 [50] |
| TiNbN (AISI 316L steel) | Arc evaporation (PVD) | 6-10 | Pin-on-disc | * Bovine serum albumin | UHMWPE | 0.13-0.4 | 1-10∙10^-3^ mm^3^/Nm (PE counter surface) | | Serro et al. 2009 [50] |
| TiCN (AISI 316L steel) | Arc evaporation (PVD) | 6-10 | Pin-on-disc | * Bovine serum albumin | UHMWPE | 0.2-0.42 | 1-10∙10^-3^ mm^3^/Nm (PE counter surface) | | Serro et al. 2009 [50] |
| nTiN (CoCr) | Magetron sputtering (PVD) |  | Simplified knee simulator | Distilled water | UHMWPE |  | 3.531 mg/5 Mc | | Oñate et al. 2001 [62] |
| TiN (cemented carbide) | Enhanced cathodic arc magnetron sputtering (PVD) |  | Ball-on-disc | Simulated body fluid | Al_2_O_3_ | 0.363 | 3.19∙10^-6^ mm^3^/Nm | | Wang et al. 2015 [64] |
| TiAlN (cemented carbide) | Enhanced cathodic arc magnetron sputtering (PVD) |  | Ball-on-disc | Simulated body fluid | Al_2_O_3_ | 0.747 | 1.64∙10^-5^ mm^3^/Nm | | Wang et al. 2015 [64] |
| Multilayered TiAlN (Ti6Al4V) | Closed field unbalanced magnetron sputter ion plating (PVD) |  | Pin-on-disc | Not disclosed | Al_2_O_3_ | 0.23-0.44 | 1.37-15∙10^-5^ mm^3^/Nm | | Yi et al. 2016 [71] |
| Nitrided Ti–Nb–Zr–Ta (TNZT) | Continuous wave fibre laser surface treatment | 249 | Pin-on-disc | Hank’s balanced salt solution | UHMWPE |  | 1.6∙10^-6^ mm^3^/Nm (nitrided TZNT) 1∙10^-6^ mm^3^/Nm (PE) | | Chan et al. 2016 [72] |
| TiC (steel) | Plasma enhanced CVD |  | Pin-on-disc | * 10% FBS in 0.9% NaCl solution | Stainless steel ball |  | 0-8∙10^-6^ mm^3^/Nm | | Vitu et al. 2008 [73] |
| CrN (CoCr) | Electron beam PVD |  | Hip simulator | Not disclosed | XLPE |  | 9.2 mm^3^/Mc | | de Villiers et al. 2015 [74] |
| CrN (cemented carbide) | Enhanced cathodic arc magnetron sputtering (PVD) |  | Ball-on-disc | Simulated body fluid | Al_2_O_3_ ball | 0.25 | 8.81∙10^-7^mm^3^/Nm | | Wang et al. 2015 [64] |
| YSZ (AISI 316-L) | Pulsed Electron Deposition | 28-60 | Ball-on-disc | * FBS | UHMWPE | 0.05-0.17 | PE wear rate: 3-13∙10^-4^ mm^3^/Nm | | Berni et al. 2017 [60] |
| YSZ (Ti) | Pulsed Plasma Deposition | 24-40 | Ball-on-disc | * 25% FBS | UHMWPE |  | PE wear: 4.8-7.3∙10^-4^ mm^3^/Nm | | Bianchi et al. 2016 [75] |
| OxZr (Zr) | Oxidation | 2.4 | Knee simulator | Deionized water | UHMWPE |  | Score based on PE damage: 104 (165 for CoCr) | | White et al. 1994 [76] |
| OxZr (Zr) | Oxidation |  | Knee simulator | * 90% Bovine serum | PE |  | PE wear: 17.2 mg/Mc | | Ezzet et al. 2012 [77] |
| TaO_2_ (Ti6Al4V) | Magnetron sputtering (PVD) |  | Pin-on-disc | Simulated body fluid | Ti6Al4V cylinders | 0.152 | 2.22 mm^3^/Nm | | Rahmati et al. 2016 [78] |
| Multilayer TaC and Ta_2_C (CoCr) | Thermal treatment in molten salts |  | Pin/ball-on-disc | * 25% vol. of bovine serum and 75% vol. of distilled water | Al_2_O_3_ ball |  | 4∙10^-7^-7∙10^-6^ mm^3^/Nm | | Balagna et al. 2014 [79] |

## Wear debris

Since the volume, size, shape and composition of the generated wear debris is an important factor in determining the local biological response and thereby potentially the implant lifespan, it should also be assessed. It has been found that PE debris generally lie in the 0.1 to 1 µm range, which has been linked to osteolysis while debris from metal on metal implants are smaller, in the nm range, and may cause cytotoxicity, necrosis and/or pseudotumours [80]–[90].

After wear test completion, the lubricant, along with any wear debris, should be collected. All proteins present in the lubricant should be degraded to avoid charging effects during characterization by scanning electron microscopy (SEM). Depending on the material, this can be done with either hydrochloric acid or an enzyme. The procedure is specified in the ISO standard ISO 17853 [91] and CEN workshop agreements [92], [93].

## Tribocorrosion

A combination of wear and corrosion mechanisms, known as tribocorrosion or wear-corrosion, can cause further mass loss of metallic materials in contact with each other. This typically occurs through three different mechanisms; removal of the passivating layer during sliding contact, galvanic attack of the substrate and galvanic attack of the counter surface. During the first mechanism the passive layer is continuously removed and reformed and a galvanic attack on the metal substrate will lead to blistering and cracking. Finally a galvanic attack of the counter surface will lead to a roughening of the counter surface and subsequently increased abrasive wear of the passivating layer [94].

The first type, removal of a passivating layer, is mitigated by coating the metal with a ceramic, thereby reducing the risk for a galvanic attack of the substrate. A galvanic attack of the counter surface can at best be reduced by avoiding galvanic coupling of the sliding surfaces.

# Biocompatibility

Biocompatibility is an implant’s ability to perform satisfactorily in the human body and in the case of joint implants it is necessary to evaluate both the material itself, in both direct and indirect contact [95], and the generated wear debris [92], [93]. The first step in assessing a material’s biocompatibility is often performed *in vitro* using cells, followed by *in vivo* studies in small to large animal models. Procedures for this are described in ISO 10993 [95]. The final assessment step is a clinical study.

The *in vitro* compatibility of the coating material is often assed using a cell line, e.g. fibroblasts, that is exposed to the material or extracts from the material followed by a viability evaluation such as an MTT (3-(4,5-dimethylthiazol-2-yl)-2,5-diphenyl-2H-tetrazolium bromide) or ATP (adenosine triphosphate) assay. In the MTT assay tetrazolium salt is metabolized by proliferating cells to yield a purple formazan product. The absorbance is then measured to evaluate the cell viability. The ATP assay measures the ATP in metabolically active cells by utilizing luciferase that is oxidized by ATP with light as a byproduct. Similarly, the generated wear debris can be evaluated *in vitro* by measuring the viability of cells exposed to different concentrations of particles [93]. The wear particles used are typically obtained through wear tests and then isolated using a standard protocol such as the one specified in ISO 17853 [91]. A method for isolating particles is described in CWA 17532-1 [92].

Before being considered for clinical use a new material has to be investigated using an animal model. Unlike cell studies, which investigate the effect on one, or possibly several cell types, *in vivo* studies will provide information on how the implant would perform in a complex environment. Joint implant materials have been tested in small animals such as mice, guinea pigs and rabbits as well as larger animals such as dogs [96].

While biological evaluations are complex, time consuming and costly they are of course necessary to ensure the safety and efficacy of implants ahead of clinical trials [97]–[99].

# References

[1] ISO 7206-2, “Implants for surgery — Partial and total hip joint prostheses — Part 2: Articulating surfaces made of metallic, ceramic and plastics materials,” 2015.

[2] ISO 4287, “Geometrical Product Specifications (GPS) - Surface texture: Profile method - Terms, definitions and surface texture parameters,” 1997.

[3] ISO 4288, “Geometrical product specifica- tions (GPS) – Surface texture: Profile method – Rules and proce- dures for the assessment of surface texture,” 1996.

[4] ISO 25178-604:2013, “Geometrical product specifications (GPS) - Surface texture: Areal - Part 604: Nominal characteristics of non-contact (coherence scanning interferometry) instruments,” no. 117101, pp. 1–4, 2013.

[5] A. Leyland and A. Matthews, “On the significance of the H/E ratio in wear control: A nanocomposite coating approach to optimised tribological behaviour,” *Wear*, vol. 246, no. 1–2, pp. 1–11, 2000.

[6] D. A. Rigney, “Comments of metals on the sliding wear,” *Tribol. Int.*, vol. 30, no. 5, pp. 361–367, 1997.

[7] D. A. Rigney, “The roles of hardness in the sliding behavior of materials,” *Wear*, vol. 175, no. 1–2, pp. 63–69, 1994.

[8] W. C. Oliver and G. M. Pharr, “An improved technique for determining hardness and elastic modulus using load and displacement sensing indentation experiments,” *J. Mater. Res.*, vol. 7, no. 6, pp. 1564–1583, 1992.

[9] W. C. Oliver and G. M. Pharr, “Measurement of hardness and elastic modulus by instrumented indentation: Advances in understanding and refinements to methodology,” *J. Mater. Res.*, vol. 19, no. 01, pp. 3–20, 2004.

[10] R. Saha and W. D. Nix, “Effects of the substrate on the determination of thin film mechanical properties by nanoindentation,” *Acta Mater.*, vol. 50, no. 1, pp. 23–38, Jan. 2002.

[11] ASTM E2546-07, “Standard Practice for Instrumented Indentation Testing,” 2007.

[12] ISO 14577-1, “Metallic materials – Instrumented indentation test for hardness and materials parameters – Part 1: Test method,” 2015.

[13] ISO 14577-4, “Metallic materials — Instrumented indentation test for hardness and materials parameters — Part 4: Test method for metallic and non-metallic coatings,” 2016.

[14] P. J. Burnett and D. S. Rickerby, “The relationship between hardness and scratch adhession,” *Thin Solid Films*, vol. 154, no. 1–2, pp. 403–416, Nov. 1987.

[15] P. A. Steinmann, Y. Tardy, and H. E. Hintermann, “Adhesion testing by the scratch test method: The influence of intrinsic and extrinsic parameters on the critical load,” *Thin Solid Films*, vol. 154, no. 1–2, pp. 333–349, Nov. 1987.

[16] Aesculap, “Aesculap ® Knee Arthroplasty Comparison Smith & Ne.”

[17] K. L. Mittal, “Adhesion Measurement of Thin Films,” *Electrocompon. Sci. Technol.*, vol. 3, no. 1, pp. 21–42, 1976.

[18] ASTM C1624, “Standard Test Method for Adhesion Strength and Mechanical Failure Modes of Ceramic Coatings by Quantitative Single Point Scratch Testing,” 2012.

[19] ISO 20502, “Fine ceramics (advanced ceramics, advanced technical ceramics) - Determination of adhesion of ceramic coatings by scratch testing,” 2015.

[20] ISO 26443, “Internation Standard: Fine ceramics (advanced ceramics, advanced technical ceramics) - Rockwell indentation test for evaluation of adhesion of ceramic coatings,” 2008.

[21] VDI 3198, “VDI 3198 Coating (CVD, PVD) of cold forging tools,” 2003. .

[22] G. Hunter, “Adhesion testing of oxidized zirconium,” *Trans. Soc. Biomater.*, vol. 24, 2001.

[23] J. A. Kellum, M. Song, and J. Li, “Science review: Extracellular acidosis and the immune response: Clinical and physiologic implications,” *Crit. Care*, vol. 8, no. 5, pp. 331–336, 2004.

[24] ISO 16429, “Implants for surgery — Measurements of open-circuit potential to assess corrosion behaviour of metallic implantable materials and medical devices over extended time periods,” 2004.

[25] ASTM G5, “Standard Reference Test Method for Making Potentiodynamic Anodic Polarization Measurements.”

[26] ASTM G1 - 03(2017)e1, “Standard Practice for Preparing, Cleaning, and Evaluating Corrosion Test Specimens.”

[27] A. P. D. Elfick, S. L. Smith, and A. Unsworth, “Variation in the wear rate during the life of a total hip arthroplasty: A simulator and retrieval study,” *J. Arthroplasty*, vol. 15, no. 7, pp. 901–908, 2000.

[28] B. Boyer, T. Neri, J. Geringer, A. Di Iorio, R. Philippot, and F. Farizon, “Long-term wear of dual mobility total hip replacement cups: explant study,” *Int. Orthop.*, vol. 42, no. 1, pp. 41–47, 2018.

[29] S. C. Scholes, B. J. Hunt, V. M. Richardson, D. J. Langton, E. Smith, and T. J. Joyce, “Explant analysis of the Biomet Magnum/ReCap metal-on-metal hip joint,” *Bone Jt. Res.*, vol. 6, no. 2, pp. 113–122, 2017.

[30] American Joint Replacement Registry, “Annual Report,” 2019.

[31] Australian Orthopaedic Association National Joint Replacement Registry, “Annual Report,” 2019.

[32] Canadian Joint Replacement Registry, “Annual Report,” 2018.

[33] Danish Hip Arthroplasty Register, “Annual Report,” 2019.

[34] N. I. and the I. of M. National Joint Registry for England, Wales, “Annual Report,” 2019.

[35] Norwegian Arthroplasty Register, “Annual report,” 2019.

[36] Swedish Hip Arthroplasty Register, “Annual report,” 2018.

[37] Swedish Knee Arthroplasty Register, “Annual Report,” 2019.

[38] The New Zealand Joint Registry, “Annual Report,” 2018.

[39] WHO, “Annex B. Tables of Health Statistics by Country, WHO Region and Globally,” *World Heal. Stat.*, no. April, pp. 103–120, 2016.

[40] J. L. Tipper *et al.*, “Isolation and characterization of UHMWPE wear particles down to ten nanometers in size from in vitro hip and knee joint simulators,” *J. Biomed. Mater. Res. Part A*, vol. 79, no. 4, pp. 473–80, 2006.

[41] C. L. Bladen *et al.*, “Analysis of wear, wear particles, and reduced inflammatory potential of vitamin e ultrahigh-molecular-weight polyethylene for use in total joint replacement,” *J. Biomed. Mater. Res. - Part B Appl. Biomater.*, vol. 101 B, no. 3, pp. 458–466, 2013.

[42] ASTM F732, “Standard Test Method for Wear Testing of Polymeric Materials Used in Total Joint,” no. Reapproved 2006, 2000.

[43] ASTM F1714-96, “Standard Guide for Gravimetric Wear Assessment of Prosthetic Knee Designs,” no. Reapproved 2008, 1996.

[44] ISO 14242-1, “Implants for surgery — Wear of total hip-joint prostheses — Part 1: Loading and displacement parameters for wear-testing machines and corresponding environmental conditions for test,” 2014.

[45] ISO 14243-1, “Implants for surgery — Wear of total knee-joint prostheses — Part 1: Loading and displacement parameters for wear-testing machines with load control and corresponding environmental conditions for test,” 1999.

[46] ISO/TR 9325, “Implants for surgery -- Partial and total hip joint prostheses -- Recommendations for simulators for evaluation of hip joint prostheses,” 1989.

[47] EN 1071-12, “Advanced technical ceramics – Methods of test for ceramic coatings – Part 12: Reciprocating wear test,” 2001.

[48] ISO 20808, “Fine ceramics (advanced ceramics, advanced technical ceramics) - Determination of friction and wear characteristics of monolithic ceramics by ball-on-disc method,” 2016.

[49] EN 1071-13, “Advanced technical ceramics. Methods of test for ceramic coatings - Part 13: Determination of wear rate by the pin-on-disk method,” 2010.

[50] A. P. Serro *et al.*, “A comparative study of titanium nitrides, TiN, TiNbN and TiCN, as coatings for biomedical applications,” *Surf. Coatings Technol.*, vol. 203, no. 24, pp. 3701–3707, 2009.

[51] F. Guo, Z. Zhou, M. Hua, and G. Dong, “Effect of aqueous solution and load on the formation of DLC transfer layer against Co-Cr-Mo for joint prosthesis,” *J. Mech. Behav. Biomed. Mater.*, vol. 49, pp. 12–22, 2015.

[52] ISO 14243-3, “Implants for surgery — Wear of total knee-joint prostheses — Part 3: Loading and displacement parameters for wear-testing machines with displacement control and corresponding environmental conditions for test,” 1999.

[53] ISO 14242-4, “Implants for surgery — Wear of total hip-joint prostheses — Part 4: Testing hip prostheses under variations in component positioning which results in direct edge loading Implants,” 2018.

[54] ISO 14242-3, “Implants for surgery — Wear of total hip- joint prostheses — Part 3: Loading and displacement parameters for orbital bearing type wear testing machines and corresponding environmental conditions for test,” 2000.

[55] R. Willing, “Comparing damage on retrieved total elbow replacement bushings with lab worn specimens subjected to varied loading conditions,” *J. Orthop. Res.*, pp. 1–9, 2018.

[56] S. L. Smith, E. Kennard, and T. J. Joyce, “Shoulder Simulator Wear Test of Five Contemporary Total Shoulder Prostheses With Three Axes of Rotation and Sliding Motion,” *Biotribology*, vol. 13, no. September 2017, pp. 36–41, 2018.

[57] W. L. Walter, G. M. Insley, W. K. Walter, and M. A. Tuke, “Edge loading in third generation alumina ceramic-on-ceramic bearings: Stripe wear,” *J. Arthroplasty*, vol. 19, no. 4, pp. 402–413, 2004.

[58] D. E. Lunn, E. De Pieri, G. J. Chapman, M. E. Lund, A. C. Redmond, and S. J. Ferguson, “Current Preclinical Testing of New Hip Arthroplasty Technologies Does Not Reflect Real-World Loadings: Capturing Patient-Specific and Activity-Related Variation in Hip Contact Forces,” *J. Arthroplasty*, vol. 35, no. 3, pp. 877–885, 2020.

[59] S. C. Scholes, “Physics in Medicine & Biology Related content A frictional study of total hip joint replacements,” *Phys. Med. Biol.*, vol. 45, p. 3721, 2000.

[60] M. Berni *et al.*, “Effects of working gas pressure on zirconium dioxide thin film prepared by pulsed plasma deposition: roughness, wettability, friction and wear characteristics,” *J. Mech. Behav. Biomed. Mater.*, vol. 72, no. February, pp. 200–208, 2017.

[61] G. Thorwarth *et al.*, “Tribological behavior of DLC-coated articulating joint implants,” *Acta Biomater.*, vol. 6, no. 6, pp. 2335–2341, 2010.

[62] J. I. Oñate *et al.*, “Wear reduction effect on ultra-high-molecular-weight polyethylene by application of hard coatings and ion implanation on cobalt chromium ally, as measured in a knee wear simulation machine,” *Surf. Coatings Technol.*, vol. 142–144, pp. 1056–1062, 2001.

[63] D. P. Dowling *et al.*, “Evaluation of diamond-like carbon-coated orthopaedic implants,” vol. 9635, pp. 5–8, 1997.

[64] Q. Wang *et al.*, “Comparison of tribological and electrochemical properties of TiN, CrN, TiAlN and a-C:H coatings in simulated body fluid,” *Mater. Chem. Phys.*, vol. 158, pp. 74–81, 2015.

[65] M. M. Maru *et al.*, “The High performance of nanocrystalline CVD diamond coated hip joints in wear simulator test,” *J. Mech. Behav. Biomed. Mater.*, vol. 49, pp. 175–185, 2015.

[66] M. Pettersson *et al.*, “Mechanical and tribological behavior of silicon nitride and silicon carbon nitride coatings for total joint replacements.,” *J. Mech. Behav. Biomed. Mater.*, vol. 25, pp. 41–7, Sep. 2013.

[67] J. Olofsson *et al.*, “Fabrication and evaluation of SixNy coatings for total joint replacements.,” *J. Mater. Sci. Mater. Med.*, vol. 23, no. 8, pp. 1879–89, Aug. 2012.

[68] L. C. Filho *et al.*, “The effect of coating density on functional properties of SiNx coated implants,” *Materials (Basel).*, vol. 12, no. 20, pp. 11–14, 2019.

[69] L. C. Filho *et al.*, “The effect of N, C, Cr, and Nb content on silicon nitride coatings for joint applications,” *Materials (Basel).*, vol. 13, no. 8, 2020.

[70] L. Filho, S. Schmidt, K. Leifer, H. Engqvist, H. Högberg, and C. Persson, “Towards functional silicon nitride coatings for joint replacements,” *Coatings*, vol. 9, no. 2, pp. 1–10, 2019.

[71] P. Yi, L. Peng, and J. Huang, “Multilayered TiAlN films on Ti6Al4V alloy for biomedical applications by closed field unbalanced magnetron sputter ion plating process,” *Mater. Sci. Eng. C*, vol. 59, pp. 669–676, 2016.

[72] C. W. Chan *et al.*, “Enhancement of wear and corrosion resistance of beta titanium alloy by laser gas alloying with nitrogen,” *Appl. Surf. Sci.*, vol. 367, pp. 80–90, 2016.

[73] T. Vitu *et al.*, “Surface & Coatings Technology Structure and tribology of biocompatible Ti – C : H coatings,” *Surf. Coat. Technol.*, vol. 202, no. 22–23, pp. 5790–5793, 2008.

[74] D. de Villiers, A. Traynor, S. N. Collins, S. Banfield, J. Housden, and J. C. Shelton, “Chromium nitride coating for large diameter metal-on-polyethylene hip bearings under extreme adverse hip simulator conditions,” *Wear*, vol. 328–329, pp. 363–368, 2015.

[75] M. Bianchi *et al.*, “Surface morphology, tribological properties and in vitro biocompatibility of nanostructured zirconia thin films,” *J. Mater. Sci. Mater. Med.*, vol. 27, no. 5, pp. 1–10, 2016.

[76] S. E. White, L. A. Whiteside, D. S. McCarthy, M. Anthony, and R. A. Poggie, “Simulated knee wear with cobalt chromium and oxidized zirconium knee femoral components.pdf.” 1994.

[77] K. A. Ezzet, J. C. Hermida, N. Steklov, and D. D. D. Lima, “Wear of Polyethylene Against Oxidized Zirconium Femoral Components Effect of Aggressive Kinematic Conditions and Malalignment in Total Knee Arthroplasty,” *J. Arthroplasty*, vol. 27, no. 1, pp. 116–121, 2012.

[78] B. Rahmati, A. A. D. Sarhan, W. J. Basirun, and W. A. B. W. Abas, “Ceramic tantalum oxide thin film coating to enhance the corrosion and wear characteristics of Ti-6Al-4V alloy,” *J. Alloys Compd.*, 2016.

[79] C. Balagna, M. G. Faga, and S. Spriano, “Tribological behavior of a Ta-based coating on a Co–Cr–Mo alloy,” *Surf. Coatings Technol.*, Jul. 2014.

[80] J. H. Dumbleton, M. T. Manley, and A. A. Edidin, “A literature review of the association between wear rate and osteolysis in total hip arthroplasty,” *J. Arthroplasty*, vol. 17, no. 5, pp. 649–661, 2002.

[81] G. M. Keegan, I. D. Learmonth, and C. P. Case, “Orthopaedic metals and their potential toxicity in the arthroplasty patient: A REVIEW OF CURRENT KNOWLEDGE AND FUTURE STRATEGIES,” *J. Bone Jt. Surg. - Br. Vol.*, vol. 89-B, no. 5, pp. 567–573, 2007.

[82] M. Huber, G. Reinisch, G. Trettenhahn, K. Zweymüller, and F. Lintner, “Presence of corrosion products and hypersensitivity-associated reactions in periprosthetic tissue after aseptic loosening of total hip replacements with metal bearing surfaces,” *Acta Biomater.*, vol. 5, no. 1, pp. 172–180, 2009.

[83] P. Korovessis, G. Petsinis, M. Repanti, and T. Repantis, “Metallosis After Contemporary Metal-on-Metal Total Hip Arthroplasty,” *J. Bone Jt. Surg.*, vol. 88, no. 6, pp. 1183–1191, 2006.

[84] E. Ingham and J. Fisher, “The role of macrophages in osteolysis of total joint replacement,” *Biomaterials*, vol. 26, no. 11, pp. 1271–1286, 2005.

[85] J. R. Campbell and M. P. Estey, “Metal release from hip prostheses: Cobalt and chromium toxicity and the role of the clinical laboratory,” *Clin. Chem. Lab. Med.*, vol. 51, no. 1, pp. 213–220, 2013.

[86] J. J. Jacobs, K. a Roebuck, M. Archibeck, N. J. Hallab, and T. T. Glant, “Osteolysis: basic science.,” *Clin. Orthop. Relat. Res.*, no. 393, pp. 71–7, 2001.

[87] N. Sugano *et al.*, “Nationwide investigation into adverse tissue reactions to metal debris after metal-on-metal total hip arthroplasty in Japan,” *J. Orthop. Sci.*, vol. 19, no. 1, pp. 85–89, 2014.

[88] N. J. Hallab and J. J. Jacobs, “Biologic effects of implant debris,” *Bull. NYU Hosp. Jt. Dis.*, vol. 67, no. 2, pp. 182–188, 2009.

[89] P. F. Doorn, P. A. Campbell, J. Worrall, P. D. Benya, H. A. McKellop, and H. C. Amstutz, “Metal wear particle characterization from metal on metal total hip replacements: Transmission electron microscopy study of periprosthetic tissues and isolated particles,” *J. Biomed. Mater. Res.*, vol. 42, no. 1, pp. 103–111, 1998.

[90] C. Fleury *et al.*, “Effect of cobalt and chromium ions on human MG-63 osteoblasts in vitro: Morphology, cytotoxicity, and oxidative stress,” *Biomaterials*, vol. 27, no. 18, pp. 3351–3360, 2006.

[91] ISO 17853:2011, “Wear of implant materials — Polymer and metal wear particles — Isolation and characterization,” 2011.

[92] CWA 17253-1, “Joint implants - Part 1: Novel methods for isolating wear particles from joint replacements and related devices This,” 2018.

[93] CWA 17253-2, “Joint implants - Part 2: Tiered toolkit approach to evaluate the biological impact of wear particles from joint replacements and related devices,” 2018.

[94] P. A. Dearnley and G. Aldrich-Smith, “Corrosion-wear mechanisms of hard coated austenitic 316L stainless steels,” *Wear*, vol. 256, no. 5, pp. 491–499, 2004.

[95] ISO 10993, “Biological evaluation of medical devices,” 2009.

[96] Y. H. An and R. J. Friedman, “Animal models of orthopedic implant infection,” *J. Investig. Surg.*, vol. 11, no. 2, pp. 139–146, 1998.

[97] S. J. M. Breugem *et al.*, “Evaluation of 1031 primary titanium nitride coated mobile bearing total knee arthroplasties in an orthopedic clinic,” vol. 8, no. 12, pp. 922–928, 2017.

[98] A. Postler, F. Beyer, C. Lützner, E. Tille, and J. Lützner, “Similar outcome during short-term follow-up after coated and uncoated total knee arthroplasty: a randomized controlled study,” *Knee Surgery, Sport. Traumatol. Arthrosc.*, vol. 26, no. 11, pp. 3459–3467, 2018.

[99] R. P. Hove, R. M. Brohet, B. J. Royen, and P. A. Nolte, “No clinical benefit of titanium nitride coating in cementless mobile-bearing total knee arthroplasty,” *Knee Surgery, Sport. Traumatol. Arthrosc.*, vol. 23, no. 6, pp. 1833–1840, 2015.
